# Supplementary material for: Economic evaluation protocol of a short, all-oral bedaquiline-containing regimen for the treatment of rifampicin-resistant tuberculosis from the STREAM trial
Source: BMJ Open. 2020 Dec 20;10(12):e042390. doi: 10.1136/bmjopen-2020-042390 (PMC7754660; doi:10.1136/bmjopen-2020-042390)
Supplement: Supplementary data [file bmjopen-2020-042390supp001.pdf]

-Supplementary material-

Annex 1- CHEERS Checklist

Section	Item No	Recommendation	Reported on page No/line No
**Title and Abstract**			
**Title**	1	Identify the study as an economic evaluation or use more specific terms such as "cost-effectiveness analysis", and describe the interventions compared.	Title is: Economic evaluation of a short, all-oral bedaquiline-containing regimen for the treatment of rifampicin-resistant tuberculosis from the STREAM trial
**Abstract**	2	Provide a structured summary of objectives, perspective, setting, methods (including study design and inputs), results (including base case and uncertainty analyses), and conclusions.	N/A as it is an analysis plan
**Introduction**			
**Background and objectives**	3	Provide an explicit statement of the broader context for the study.<br><br>Present the study question and its relevance for health policy or practice decisions.	Covered in Background and Objectives sections
**Methods**			
**Target population and subgroups**	4	Describe characteristics of the base case population and subgroups analyzed, including why they were chosen.	Covered in the Methods and Analysis section
**Setting and location**	5	State relevant aspects of the system(s) in which the decision(s) need(s) to be made.	Covered in the Methods and Analysis section
**Study perspective**	6	Describe the perspective of the study and relate this to the costs being evaluated.	Covered in the Methods and Analysis section
**Comparators**	7	Describe the interventions or strategies being compared and state why they were chosen.	Covered in the Methods and Analysis section
**Time horizon**	8	State the time horizon(s) over which costs and consequences are being evaluated and say why appropriate.	Covered in the Methods and Analysis section
**Discount rate**	9	Report the choice of discount rate(s) used for costs and outcomes and say why appropriate.	In the Methods section, Health system resource use and cost sub-heading
**Choice of health outcomes**	10	Describe what outcomes were used as the measure(s) of benefit in the evaluation and their relevance for the type of analysis performed.	Covered in the Methods and Analysis section
**Measurement of effectiveness**	11a	*Single study-based estimates*: Describe fully the design features of the single effectiveness study and why the single study was a sufficient source of clinical effectiveness data.	Reference to the clinical paper; Covered in the Methods and Analysis section
	11b	*Synthesis-based estimates*: Describe fully the methods used for identification of included studies and synthesis of clinical effectiveness data.	N/A

**Measurement and valuation of preference-based outcomes**	12	If applicable, describe the population and methods used to elicit preferences for outcomes.	N/A
**Estimating resources and costs**	13a	*Single study-based economic evaluation*: Describe approaches used to estimate resource use associated with the alternative interventions. Describe primary or secondary research methods for valuing each resource item in terms of its unit cost. Describe any adjustments made to approximate to opportunity costs.	Fully described in Methods and Analysis section
	13b	*Model-based economic evaluation*: Describe approaches and data sources used to estimate resource use associated with model health states. Describe primary or secondary research methods for valuing each resource item in terms of its unit cost. Describe any adjustments made to approximate to opportunity costs.	N/A
**Currency, price date, and conversion**	14	Report the dates of the estimated resource quantities and unit costs. Describe methods for adjusting estimated unit costs to the year of reported costs if necessary. Describe methods for converting costs into a common currency base and the exchange rate.	Dates of the estimated resource quantities and unit costs not reported as this is a protocol. Methods for adjusting the unit costs and converting costs into a common currency are covered in the Methods and Analysis section, after the Patient costs sub-heading.
**Choice of model**	15	Describe and give reasons for the specific type of decision analytical model used. Providing a figure to show model structure is strongly recommended.	N/A as not a model-based evaluation
**Assumptions**	16	Describe all structural or other assumptions underpinning the decision-analytical model.	N/A as not a model-based evaluation
**Analytical methods**	17	Describe all analytical methods supporting the evaluation. This could include methods for dealing with skewed, missing, or censored data; extrapolation methods; methods for pooling data; approaches to validate or make adjustments (such as half cycle corrections) to a model; and methods for handling population heterogeneity and uncertainty.	Fully covered in the Methods and Analysis section, in the Missing data, Statistical analysis and Sensitivity analyses sub-sections.
**Results**			
**Study parameters**	18	Report the values, ranges, references, and, if used, probability distributions for all parameters. Report reasons or sources for distributions used to represent uncertainty where appropriate. Providing a table to show the input values is strongly recommended.	N/A as this is a study protocol, but these will be presented in the main paper as stated in this protocol
**Incremental costs and outcomes**	19	For each intervention, report mean values for the main categories of estimated costs and outcomes of interest, as well as mean differences between the comparator groups. If applicable, report incremental cost-effectiveness ratios.	N/A as this is a study protocol, but these will be presented in the main paper as stated in this protocol

**Characterizing uncertainty**	**20a**	*Single study-based economic evaluation*: Describe the effects of sampling uncertainty for the estimated incremental cost and incremental effectiveness parameters, together with the impact of methodological assumptions (such as discount rate, study perspective).	Methods and Analysis section of the protocol-Sensitivity analyses sub-heading.
	20b	*Model-based economic evaluation*: Describe the effects on the results of uncertainty for all input parameters, and uncertainty related to the structure of the model and assumptions.	N/A
**Characterizing heterogeneity**	21	If applicable, report differences in costs, outcomes, or cost-effectiveness that can be explained by variations between subgroups of patients with different baseline characteristics or other observed variability in effects that are not reducible by more information.	Costs and outcomes will be presented separately for each country

**Discussion**			
**Study findings, limitations, generalizability, and current knowledge**	22	Summarise key study findings and describe how they support the conclusions reached. Discuss limitations and the generalizability of the findings and how the findings fit with current knowledge.	Discussion about the strengths and limitations in the Discussion section; the key findings and their generalizability will be presented in the paper.
**Other**			
**Source of funding**	23	Describe how the study was funded and the role of the funder in the identification, design, conduct, and reporting of the analysis. Describe other non-monetary sources of support.	Acknowledgements
**Conflicts of interest**	24	Describe any potential for conflict of interest of study contributors in accordance with journal policy. In the absence of a journal policy, we recommend authors comply with International Committee of Medical Journal Editors recommendations.	Acknowledgements

Annex 2- Value sets to be used

Independent variables of the model	C-TTO Tobit model censored at −1			DCE conditional logistic model rescaled			Hybrid model censored C-TTO values at −1 (final value set)		
	Coeff.	(SE)	*p* value	Coeff.	(SE)	*p* value	Coeff.	(SE)	*p* value
Mobility (MO)									
No problems to slight problems	0.088	(0.015)	0.000	0.139	(0.015)	0.000	0.119	(0.008)	0.000
Slight problems to moderate problems	0.086	(0.017)	0.000	0.080	(0.017)	0.000	0.073	(0.011)	0.000
Moderate problems to severe problems	0.250	(0.019)	0.000	0.196	(0.016)	0.000	0.218	(0.013)	0.000
Severe problems to unable	0.170	(0.018)	0.000	0.219	(0.018)	0.000	0.203	(0.012)	0.000
Self-care (SC)									
No problems to slight problems	0.085	(0.014)	0.000	0.101	(0.016)	0.000	0.101	(0.007)	0.000
Slight problems to moderate problems	0.056	(0.018)	0.002	0.038	(0.018)	0.032	0.039	(0.010)	0.000
Moderate problems to severe problems	0.128	(0.018)	0.000	0.085	(0.019)	0.000	0.108	(0.013)	0.000
Severe problems to unable	0.035	(0.016)	0.030	0.097	(0.017)	0.000	0.068	(0.012)	0.000
Usual activities (UA)									
No problems to slight problems	0.071	(0.015)	0.000	0.092	(0.016)	0.000	0.090	(0.006)	0.000
Slight problems to moderate problems	0.106	(0.017)	0.000	0.051	(0.017)	0.003	0.066	(0.011)	0.000
Moderate problems to severe problems	0.137	(0.019)	0.000	0.154	(0.017)	0.000	0.145	(0.013)	0.000
Severe problems to unable	0.061	(0.018)	0.001	0.091	(0.017)	0.000	0.084	(0.013)	0.000
Pain/discomfort (PD)									
No problems to slight problems	0.089	(0.013)	0.000	0.081	(0.016)	0.000	0.086	(0.006)	0.000
Slight problems to moderate problems	0.007	(0.019)	0.721	0.012	(0.018)	0.513	0.009	(0.011)	0.395
Moderate problems to severe problems	0.135	(0.018)	0.000	0.085	(0.017)	0.000	0.103	(0.013)	0.000
Severe problems to extreme problems	0.024	(0.019)	0.211	0.053	(0.018)	0.003	0.048	(0.013)	0.000
Anxiety/depression (AD)									
No problems to slight problems	0.079	(0.014)	0.000	0.050	(0.017)	0.003	0.079	(0.006)	0.000
Slight problems to moderate problems	0.055	(0.018)	0.002	0.061	(0.017)	0.000	0.055	(0.011)	0.000
Moderate problems to severe problems	0.086	(0.017)	0.000	0.114	(0.018)	0.000	0.093	(0.012)	0.000
Severe problems to extreme problems	0.062	(0.016)	0.000	0.085	(0.018)	0.000	0.078	(0.012)	0.000
Log likelihood	−6189.97			−3958.62			−9325.84		
AIC	12,421.93			7957.24			18,735.69		
BIC	12,572.19			8109.23			19,060.41		
Examples of estimated utility values									
U(21111)	0.912			0.861			0.881		
U(31111)	0.826			0.781			0.808		
U(41111)	0.576			0.585			0.590		
U(51111)	0.406			0.366			0.387		
U(12345)	0.225			0.268			0.240		
U(21231)	0.745			0.676			0.696		
U(55555)	−0.810			−0.884			−0.865		

AIC Akaike information criteria, *BIC* Bayesian information criteria, *C-TTO* composite time trade-off, *DCE* discrete choice experiments, SE standard error

Table 1- Value set to be used for India. Purba FD, Hunfeld JAM, Iskandarsyah A, et al. The Indonesian EQ-5D-5L Value Set. Pharmacoeconomics. 2017;35(11):1153-1165. doi:10.1007/s40273-017-0538-9

**Independent variables of the model**	**C-TTO OLS model**			**DCE conditional logistic model rescaled**			**Hybrid model censored C-TTO values at -1 (final value set)**		
	Coef.	(SE)	p-value	Coef.	(SE)	p-value	Coef.	(SE)	p-value
**Mobility (MO)**									
MO2	0.0047	0.014	0.729	0.4780	0.061	0.000	0.0337	0.005	0.000
MO3	0.0166	0.015	0.262	0.1138	0.071	0.110	0.0307	0.009	0.000
MO4	0.1748	0.016	0.000	0.9810	0.070	0.000	0.1632	0.010	0.000
MO5	0.1038	0.016	0.000	0.7434	0.074	0.000	0.1322	0.010	0.000
**Self-care (SC)**									
SC2	0.0036	0.013	0.785	0.2044	0.067	0.002	0.0235	0.005	0.000
SC3	0.0494	0.016	0.002	-0.0024	0.074	0.974	0.0160	0.008	0.042
SC4	0.1189	0.015	0.000	0.6849	0.078	0.000	0.1024	0.009	0.000
SC5	0.0826	0.013	0.000	0.4234	0.073	0.000	0.0804	0.009	0.000
**Usual-activities (UA)**									
UA2	0.0188	0.014	0.176	0.3470	0.063	0.000	0.0323	0.005	0.000
UA3	0.0441	0.014	0.002	-0.0391	0.071	0.579	0.0160	0.008	0.042
UA4	0.1299	0.016	0.000	0.5818	0.071	0.000	0.1091	0.009	0.000
UA5	0.0936	0.015	0.000	0.6079	0.076	0.000	0.1147	0.010	0.000
**Pain/discomfort (PD)**									
PD2	0.0140	0.013	0.266	0.4499	0.067	0.000	0.0361	0.004	0.000
PD3	0.0161	0.017	0.331	0.1090	0.073	0.136	0.0155	0.008	0.061
PD4	0.2452	0.015	0.000	1.1358	0.077	0.000	0.2187	0.010	0.000
PD5	0.1421	0.016	0.000	0.5689	0.076	0.000	0.1361	0.011	0.000
**Anxiety/depression (AD)**									
AD2	0.0111	0.014	0.428	0.2718	0.070	0.000	0.0259	0.004	0.000
AD3	0.0381	0.015	0.012	0.3516	0.072	0.000	0.0589	0.008	0.000
AD4	0.2322	0.015	0.000	1.1803	0.079	0.000	0.2139	0.009	0.000
AD5	0.1414	0.013	0.000	0.8320	0.078	0.000	0.1591	0.010	0.000
**AIC**	10587.06			6498.30			14002.09		
**BIC**	10739.33			6650.17			14336.81		
**Order of importance**									
	AD			AD			AD		
	PD			MO			PD		
	MO			PD			MO		
	UA			UA			UA		
	SC			SC			SC		

Coef. – coefficient; SE – standard error

Items with a negative coefficient (in grey) represent inconsistent items
Order of importance based on sum of disutility which is the disutility associated with level 5

Table 2- Value set to be used for Uganda and Ethiopia. Welie AG, Gebretekle GB, Stolk E, Mukuria C, Krahn MD, Enquoselassie F, Fenta TG. Valuing health state: an EQ-5D-5L value set for Ethiopians. Value Health Reg Issues. 2019;22:7–14

	Model 1 panel, random effects	Model 2 Bayesian	Model 3 M2 + random parameters	Model 4 M3 + error scaling with *t*-Student	Model 5 M4 + religion scaling	Final model M5 + DCE, censoring
Const.	0.005 (−0.010; 0.019)	Not used	Not used	Not used	Not used	Not used
MO2	0.021 (0.002; 0.039)	0.023 (0.001; 0.044)	0.058 (0.013; 0.073)	0.017 (0.014; 0.022)	0.019 (0.014; 0.023)	0.025 (0.020; 0.029)
MO3	0.012 (−0.007; 0.031)	0.016 (0.000; 0.036)	0.077 (0.021; 0.094)	0.015 (0.005; 0.026)	0.016 (0.005; 0.028)	0.034 (0.026; 0.042)
MO4	0.098 (0.077; 0.118)	0.101 (0.074; 0.129)	0.159 (0.071; 0.181)	0.101 (0.085; 0.116)	0.107 (0.090; 0.124)	0.126 (0.113; 0.141)
**MO5**	**0.262 (0.238; 0.285)**	**0.263 (0.239; 0.289)**	**0.303 (0.271; 0.330)**	**0.251 (0.228; 0.274)**	**0.267 (0.242; 0.293)**	**0.314 (0.286; 0.342)**
SC2	0.030 (0.014; 0.046)	0.037 (0.015; 0.059)	0.015 (0.003; 0.087)	0.029 (0.024; 0.034)	0.031 (0.026; 0.036)	0.031 (0.027; 0.036)
SC3	0.038 (0.017; 0.059)	0.042 (0.014; 0.071)	0.005 (0.000; 0.119)	0.037 (0.028; 0.047)	0.040 (0.029; 0.050)	0.047 (0.040; 0.055)
SC4	0.122 (0.098; 0.146)	0.116 (0.089; 0.143)	0.042 (0.027; 0.180)	0.108 (0.094; 0.123)	0.115 (0.099; 0.131)	0.111 (0.099; 0.123)
**SC5**	**0.276 (0.254; 0.298)**	**0.269 (0.244; 0.295)**	**0.242 (0.193; 0.268)**	**0.258 (0.237; 0.282)**	**0.273 (0.249; 0.299)**	**0.264 (0.243; 0.286)**
UA2	0.031 (0.014; 0.048)	0.034 (0.011; 0.058)	0.002 (0.000; 0.007)	0.033 (0.026; 0.039)	0.034 (0.028; 0.042)	0.023 (0.019; 0.027)
UA3	0.032 (0.009; 0.054)	0.041 (0.015; 0.067)	0.005 (0.000; 0.014)	0.050 (0.040; 0.060)	0.053 (0.043; 0.063)	0.040 (0.032; 0.048)
UA4	0.092 (0.070; 0.115)	0.088 (0.062; 0.115)	0.024 (0.010; 0.038)	0.104 (0.091; 0.117)	0.110 (0.095; 0.125)	0.097 (0.087; 0.107)
**UA5**	**0.186 (0.167; 0.206)**	**0.183 (0.157; 0.209)**	**0.180 (0.161; 0.201)**	**0.180 (0.161; 0.200)**	**0.190 (0.169; 0.212)**	**0.205 (0.188; 0.224)**
PD2	0.028 (0.012; 0.044)	0.033 (0.012; 0.054)	0.041 (0.028; 0.054)	0.025 (0.021; 0.028)	0.026 (0.022; 0.030)	0.030 (0.026; 0.034)
PD3	0.034 (0.014; 0.053)	0.035 (0.007; 0.063)	0.053 (0.036; 0.071)	0.030 (0.022; 0.039)	0.032 (0.022; 0.041)	0.050 (0.043; 0.058)
PD4	0.229 (0.208; 0.251)	0.228 (0.204; 0.254)	0.253 (0.224; 0.276)	0.223 (0.208; 0.239)	0.235 (0.217; 0.253)	0.261 (0.244; 0.280)
**PD5**	**0.467 (0.440; 0.494)**	**0.473 (0.446; 0.499)**	**0.490 (0.464; 0.518)**	**0.492 (0.463; 0.520)**	**0.519 (0.485; 0.555)**	**0.575 (0.538; 0.613)**
AD2	0.024 (0.006; 0.041)	0.032 (0.010; 0.054)	0.049 (0.015; 0.061)	0.019 (0.016; 0.023)	0.020 (0.017; 0.024)	0.018 (0.015; 0.021)
AD3	0.034 (0.011; 0.056)	0.033 (0.006; 0.058)	0.085 (0.038; 0.101)	0.037 (0.026; 0.049)	0.039 (0.027; 0.052)	0.029 (0.022; 0.037)
AD4	0.114 (0.094; 0.135)	0.114 (0.088; 0.139)	0.160 (0.116; 0.181)	0.119 (0.106; 0.132)	0.126 (0.113; 0.142)	0.108 (0.097; 0.119)
**AD5**	**0.224 (0.203; 0.244)**	**0.226 (0.201; 0.251)**	**0.176 (0.153; 0.231)**	**0.211 (0.194; 0.229)**	**0.223 (0.204; 0.243)**	**0.232 (0.213; 0.252)**
Deviance	61.2% (R^2 used instead)	11,866	−777	−13,781	−13,780	−9215
DIC		11,886	2597	−9704	−9704	−9215[a]
PSRF	n.a.	All <1.01	Maximum = 15	All <1.01	All <1.01	All <1.01
Maximum *u* (not 11111)	0.983	0.984	0.998	0.985	0.984	0.982
u (22222)	0.862	0.841	0.834	0.877	0.870	0.873
u (33333)	0.847	0.833	0.775	0.830	0.821	0.800
u (44444)	0.340	0.352	0.361	0.345	0.307	0.296
u (55555)	− 0.420	− 0.415	− 0.391	− 0.392	− 0.471	− 0.590
% states $u < 0$	2.85	2.88	2.69	2.78	4.26	6.66
Dimension order	PD, SC, MO, AD, UA	PD, SC, MO, AD, UA	PD, MO, SC, UA, AD	PD, SC, MO, AD, UA	PD, SC, MO, AD, UA	PD, MO, SC, AD, UA
Levels consistency	MO3 < MO2	MO3 < MO2	SC3 < SC2	MO3 < MO2	MO3 < MO2	Consistent

AD anxiety/depression, *DCE* discrete choice experiment, *DIC* deviance information criterion, *M* model, *MO* mobility, *n.a. PD* pain/discomfort, *PSRF* potential scale reduction factor, *SC* self-care, *u* utility, *UA* usual activities

[a]Failed to calculate penalty in JAGS ("support of observed nodes is not fixed")

Table 3- Value set to be used for Moldova. Golicki, D., Jakubczyk, M., Graczyk, K. et al. Valuation of EQ-5D-5L Health States in Poland: the First EQ-VT-Based Study in Central and Eastern Europe. PharmacoEconomics 37, 1165–1176 (2019). https://doi.org/10.1007/s40273-019-00811-7

Annex 3- COVID19 diary

COVID19 diary

-to be completed by focal health economists at each site-

Epidemiology of the Epidemic

- First case notification date

Details of policies declared by central/federal/state government that potentially restrict "Normal" daily life. Date implemented/Details of policy/Date lifted

- Lockdown start date
- Specific restrictions- what's the rule of going outside the house? What's the rule for going out for work?
- Law enforcement- are people being fined for going out?

- Are entertainment places open (cinemas, theatres shopping centres)? Are cricket, football, etc. competitions still taking place? If not, when were these stopped?
- Lockdown end date

Impact on daily life (descriptive/opinion) behavioural picture

- Country’s general perception regarding COVID19- are they scared, complaint with the rules, are they indifferent
- Can you find basic supplies in the markets/supermarkets? Rice, bread? Is there a price increase amongst basic supplies?
- Are people living with their families during the lockdown? Have they travelled to their home town/village during the lockdown?
- Any shortage in drug supplies?
- Anything else you would like to report, that would influence the patients’ income and their quality of life?
