# Supplementary material for: Economic evaluation protocol of a short, all-oral bedaquiline-containing regimen for the treatment of rifampicin-resistant tuberculosis from the STREAM trial
Source: BMJ Open. 2020 Dec 20;10(12):e042390. doi: 10.1136/bmjopen-2020-042390 (PMC7754660; doi:10.1136/bmjopen-2020-042390)
Supplement: Author's manuscript [file bmjopen-2020-042390.draft_revisions.pdf]

BMJ Open

BMJ Open is committed to open peer review. As part of this commitment we make the peer review history of every article we publish publicly available.

When an article is published we post the peer reviewers' comments and the authors' responses online. We also post the versions of the paper that were used during peer review. These are the versions that the peer review comments apply to.

The versions of the paper that follow are the versions that were submitted during the peer review process. They are not the versions of record or the final published versions. They should not be cited or distributed as the published version of this manuscript.

BMJ Open is an open access journal and the full, final, typeset and author-corrected version of record of the manuscript is available on our site with no access controls, subscription charges or pay-per-view fees (http://bmjopen.bmj.com).

If you have any questions on BMJ Open's open peer review process please email info.bmjopen@bmj.com

BMJ Open

Economic evaluation of a short, all-oral bedaquiline-containing regimen for the treatment of rifampicin-resistant tuberculosis from the STREAM trial

Journal:	*BMJ Open*
Manuscript ID	bmjopen-2020-042390
Article Type:	Protocol
Date Submitted by the Author:	03-Jul-2020
Complete List of Authors:	Rosu, Laura; Liverpool School of Tropical Medicine, Centre for Applied Health Research and Delivery & Department of Clinical Sciences<br>Madan, Jason; University of Warwick, Warwick Medical School<br>Worrall, E; Liverpool School of Tropical Medicine,<br>Tomeny, Ewan; Liverpool School of Tropical Medicine, Centre for Applied Health Research and Delivery & Department of Clinical Sciences<br>Squire, Bertie; Liverpool school of tropical medicine, Centre for Applied Health Research and Delivery & Department of Clinical Sciences
Keywords:	Tuberculosis < INFECTIOUS DISEASES, Economics < TROPICAL MEDICINE, Health economics < HEALTH SERVICES ADMINISTRATION & MANAGEMENT

SCHOLARONE™
Manuscripts

I, the Submitting Author has the right to grant and does grant on behalf of all authors of the Work (as defined in the below author licence), an exclusive licence and/or a non-exclusive licence for contributions from authors who are: i) UK Crown employees; ii) where BMJ has agreed a CC-BY licence shall apply, and/or iii) in accordance with the terms applicable for US Federal Government officers or employees acting as part of their official duties; on a worldwide, perpetual, irrevocable, royalty-free basis to BMJ Publishing Group Ltd ("BMJ") its licensees and where the relevant Journal is co-owned by BMJ to the co-owners of the Journal, to publish the Work in this journal and any other BMJ products and to exploit all rights, as set out in our licence.

The Submitting Author accepts and understands that any supply made under these terms is made by BMJ to the Submitting Author unless you are acting as an employee on behalf of your employer or a postgraduate student of an affiliated institution which is paying any applicable article publishing charge ("APC") for Open Access articles. Where the Submitting Author wishes to make the Work available on an Open Access basis (and intends to pay the relevant APC), the terms of reuse of such Open Access shall be governed by a Creative Commons licence – details of these licences and which Creative Commons licence will apply to this Work are set out in our licence referred to above.

Other than as permitted in any relevant BMJ Author's Self Archiving Policies, I confirm this Work has not been accepted for publication elsewhere, is not being considered for publication elsewhere and does not duplicate material already published. I confirm all authors consent to publication of this Work and authorise the granting of this licence.

For peer review only

Economic evaluation of a short, all-oral bedaquiline-containing regimen for the treatment of rifampicin-resistant tuberculosis from the STREAM trial

-Preliminary analysis plan-

Laura Rosu[1], Jason J Madan[2], Eve Worrall[3], Ewan Tomeny[1], S Bertel Squire[1] and the STREAM Study Health Economic Evaluation Collaborators

[1] Centre for Applied Health Research and Delivery, Liverpool School of Tropical Medicine, Liverpool, UK

[2] Warwick Medical School, University of Warwick, Coventry, UK

[3] Vector Biology, Liverpool School of Tropical Medicine, Liverpool, UK

Corresponding author: Laura Rosu, laura.rosu@lstmed.ac.uk

Keywords: tuberculosis, multi-drug resistant TB, cost-effectiveness analysis; cost-utility analysis; economic evaluation; short multi-drug resistant treatment; bedaquiline TB treatment; applied health research

Abstract

Introduction

A December 2019 WHO rapid communication recommended the use of nine-month, all-oral regimens for treating multi-drug resistant tuberculosis (MDR-TB). Besides the clinical benefits, they are thought to be less costly than the injectable-containing regimens, for both the patient and the health system. STREAM is the first randomised controlled trial with an economic evaluation to compare all-oral and injectable-containing 9-11 month MDR-TB treatment regimens.

Methods and Analysis

Health system costs of delivering a nine-month, injectable containing regimen and a nine-month all oral regimen will be collected in Ethiopia, India, Moldova, and Uganda, using ‘bottom-up’ and ‘top-down’ costing approaches. Patient-costs will be collected using questionnaires that have been developed based on the STOP-TB questionnaire. The primary objective of the study is to estimate the cost-utility of the two regimens, from a health system perspective. Secondary objectives include estimating the cost-utility from a societal perspective as well as evaluating the cost-effectiveness of the regimens, using both health

For peer review only

system and societal perspectives. The effect measure for the cost-utility analysis will be the QALY, while the effect measure for the cost-effectiveness analysis will be the efficacy outcome from the clinical trial.

Ethics and dissemination

The study has been evaluated and approved by the Ethics Advisory Group of the *International Union Against Tuberculosis and Lung Disease* and also approved by ethics committees in all participating countries. All participants have provided written informed consent. The results of the economic evaluation will be published in a peer-reviewed journal.

Trial registration number: ISRCTN18148631

Strengths and limitations of this study

- The economic evaluation of STREAM will be the first study to estimate the costs incurred by both patients undergoing MDR-TB treatment and the health care system within a Phase III RCT.
- The detailed costing and analysis in four different settings will provide valuable insights into the timings and drivers of the costs associated with implementation of an all oral regimen. The study will generate important evidence needed for future policy decisions, and the shaping of targeted interventions.
- The trial setting means that the experience of participants will, in places, inevitably deviate slightly from routine practice, including the frequency of visits and their locations. Though we will attempt to separate out and eliminate such research costs in our analysis, guaranteeing no interference may not be possible.

Background

The STREAM trial is a phase III non-inferiority RCT to test the efficacy, safety and economic impact of shortened multi-drug resistant tuberculosis (MDR-TB) treatment regimens. MDR-TB is a form of tuberculosis (TB) caused by bacteria that cannot be treated with two of the most powerful, first-line anti-TB drugs, isoniazid and rifampicin. Globally, in 2017, there were a little over half a million people with TB resistant to rifampicin, and out of these 82% had MDR-TB[1].

The World Health Organization's (WHO) End TB Strategy is among the health targets of the Sustainable Development Goals. It was adopted by the World Health Assembly in 2014 with the aim of reducing TB deaths by 90% and new cases by 80% between 2015 and 2030, as well as reducing to zero the number of households incurring catastrophic costs due to TB by 2020. Currently, global TB incidence is falling at 2% per year, which is insufficient to reach

For peer review only

the 2020 milestone[2]. This means that new ways of addressing the disease must be found to meet these targets. Careful evaluation of alternative treatment strategies is vital to ensure the most effective and feasible approaches are implemented.

The December 2019 WHO rapid communication recommends the use of shorter, all-oral, bedaquiline-containing regimens for MDR-TB patients[3]. It seems that all-oral regimens, as opposed to those containing injectables, are becoming the preferred option for treatment of MDR-TB as data from the South African TB programme had suggested them to improve patient outcomes. Replacing the injectable with bedaquiline resulted in better treatment success and better adherence. Beside the clinical benefits, it is also thought that the all-oral treatment leads to lower costs from a health system and patient perspective[4]. It is therefore crucial to test these hypotheses via a randomised controlled trial (RCT) in multiple settings. Furthermore, no economic analyses of the regimen have been provided, making it difficult for policymakers to assess the economic and financial impact. STREAM is the first randomised controlled trial with an economic evaluation to compare all-oral and injectable-containing 9-11 month MDR-TB treatment regimens.

Objectives

The questions that the economic evaluation is aiming to address include:

- What are the health system costs of treating MDR-TB patients using the following regimens: a 9-month injectable regimen; a 9-month non-injectable regimen; and a 6-month injectable regimen?
- What costs do patients face during and after treatment?
- How does MDR-TB affect patients' socioeconomic situations?
- What financial coping mechanisms do patients employ?

The primary economic objective is to estimate the cost-utility of the two multi-drug resistant tuberculosis (MDR-TB) interventions, in each country, from a health system perspective. To achieve this, an economic evaluation of both the costs and consequences associated with each intervention will be conducted.

Secondary economic objectives include assessing the cost-utility of the regimens from a societal perspective and evaluating the cost-effectiveness of the regimens from both a health system and societal perspective.

The effect measure for the cost-utility analysis (CUA) will be the QALY, while the effect measure of the cost-effectiveness analysis (CEA) will be the efficacy outcome from the clinical trial i.e. favourable or unfavourable.

Methods and analysis

For peer review only

Randomised controlled trial design

Health economic data will be collected alongside the STREAM trial. Its protocol has been published elsewhere[5]. In brief, the STREAM study is an international, multi-centre, parallel-group RCT of patients with MDR-TB and patients with rifampicin resistant and isoniazid-sensitive TB. It will be assessed whether the proportion of participants on regimen C with a favourable efficacy outcome at week 76 is not less on that on regimen B i.e. C is non-inferior to B. Data will also be collected on regimen D for secondary comparisons. Treatments administered are outlined in Figure 1 and explained below:

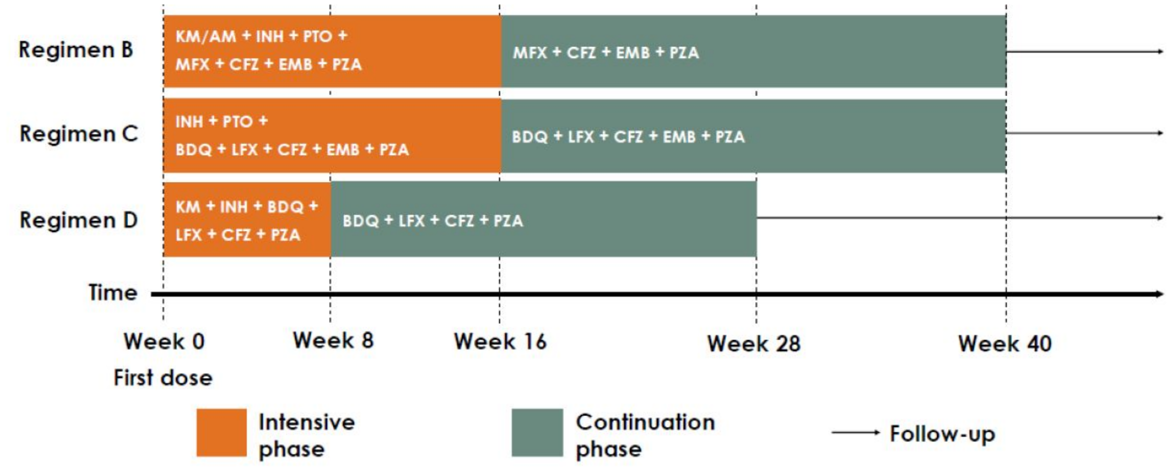

Figure 1 Treatments outline. Regimen A was dropped of the trial.

Trial recruitment started in April 2016, across 13 sites in 7 countries:

	Clinical trial sites	HE sites
**Mongolia**	National Center for Communicable Diseases, Ulaanbaatar	
**Ethiopia**	Armaeur Hansen Research Institute, Addis Ababa	x
	St. Peter's Hospital, Addis Ababa	x
**South Africa**	King Dinuzulu Hospital, Durban	
	Helen Joseph Hospital, Johannesburg	
	Empilweni TB Hospital, Port Elizabeth	
	Doris Goodwin, Pietermaritzburg	
**Moldova**	IMSP, Chiril Draganiuc, Chisinau	x
**Uganda**	Mulago Hospital, Kampala	x
**Georgia**	National Center for Tuberculosis and Lung Disease, Tbilisi	
**India**	B.J. Medical College, Ahmedabad	x
	National Institute for Research in Tuberculosis, Chennai	x
	Rajan Babu Institute for Pulmonary Medicine and Tuberculosis, Delhi	x

For peer review only - http://bmjopen.bmj.com/site/about/guidelines.xhtml

Table 1

At the start of Stage 2 randomisation was to regimen A, regimen B, regimen C and regimen D, in a ratio of 1:2:2:2, done using a web-based system managed by MRC CTU. Version 8.0 of the protocol limits randomisation to arms B and C, so patients will no longer be randomised to regimen A and D and randomisation will be in a ratio of 1:1. At least 200 patients to each of regimen B and regimen C will be randomised, across all sites. This was determined based on the assumption that the proportion of patients with a favourable efficacy outcome at week76 is 80% for regimen B and 82% for regimen C. With a non-inferiority margin of 10% and a one-sided significance level of 2.5%, 180 evaluable patients will be required in each of the two regimens to demonstrate non-inferiority. If 10% of patients will be excluded from the primary efficacy analysis population, a total of 400 patients would be required in total for regimens B and C[5].

The health economic analysis will include participants of the clinical trial in the above-mentioned sites, who are over 18 years old and fulfil the inclusion/exclusion criteria as outlined in the trial protocol. All patients in the study will be followed up until week 132, with the primary analysis conducted on data collected up to week 76.

<u>Health system resource use and costs</u>

A mixture of top-down and bottom-up approaches will be used.

Data regarding staff time and staff activities involved in the management of MDR-TB treatment for each regimen will be collected by the focal health economists in each country using a standardised questionnaire developed by the health economic team, pilot tested in all HE sites and used in the first phase of the trial[6].

A full assessment of the health system costs of delivering the MDR-TB regimens, including tests performed, consumables used, inpatient stay costs, drugs administered, and overheads will be done in each country, for each arm. Any relevant resource events will also be included. These will be collected by the focal health economists in each country using hospitals' accounting records, clinical trial CRFs and STREAM protocol, and will be costed using local unit costs where possible. Where this will not be possible, STREAM or in-country private healthcare facilities costs will be used.

The costs associated with the diagnosis and management of serious adverse events (SAEs) caused by MDR-TB or its treatment will also be included. The costing will include all tests performed, examinations, investigations, in-patient stays and medication received, as well as staff costs. Data will be collected in an event costing tool developed in Microsoft Excel by the HE trial team and the main data source will be the clinical trial CRFs.

The total health system costs for each trial arm will be estimated by summing the costs of each resource used and presented by the following cost elements, by phase:

Cost elements/Phase	**Intensive**	**Continuation**	**Follow-up**
**Inpatient stay**			

For peer review only

**Laboratory tests**			
**Medication**			
**Staff**			
**Social support**			
**Consumables**			
**Serious adverse events**			
**Overheads**			

Table 2

Capital costs extending beyond 1 year (e.g. equipment) will be annualised over their expected lifespan assuming a discount rate of 3%.

Research costs such as costs related to the pharmacokinetics study will not be collected or included in this economic evaluation. The health system costing will be done in close collaboration with the central health economic team to make sure it is sensible and evaluated with the support of a team of clinicians involved in the clinical trial. If deemed appropriate, other research costs that do not reflect usual practice will be excluded.

Patient costs

Patient costs will be collected by administering questionnaires that have been developed based on the STOP-TB questionnaire[7]. Data will be collected in two stages. First, a baseline questionnaire will capture socio-economic data of each patient before they start treatment. Then, a follow-up questionnaire capturing any changes to the socio-economic data and a patient treatment cost questionnaire will be administered every 12 weeks.

The patient costs to be collected are presented in Table 3:

Cost type	Data collection method	Analysis
Cost of attending DOTs (CostDots)	Through patient CRFs (transport and food costs data)	For each cost type category, data will be aggregated for each site and arm, to estimate the mean direct cost per visit
Costs of attending injection DOTs (CostDots)	Through patient CRFs (transport and food costs data)	
Patient cost for attending scheduled patient assessment visits (CostSVisits)	Through patient CRFs (transport and food costs data)	
Patient costs for attending unscheduled patient assessment visits (CostUVisits)	Through patient CRFs (transport and food costs data)	

For peer review only

Food supplements (CostSupp)	Through patient CRFs	Mean spend for each time point to be calculated and presented as the cumulative difference in food purchases between arms
Income loss during and after treatment	Reported by patients if willing to reveal their income at each time point; if not, working hours reported to be used as a proxy	If patients are unwilling to reveal their income, average salary values from the specific areas in each country will be used. The total lost hours will be multiplied with the hourly average wage. Total income loss during treatment and follow-up will be calculated

Table 3

The total direct cost per participant receiving MDR-TB treatment will be calculated as follows:

$$Total\ direct\ cost = (CostDots * NoVisitsD) + (CostSVisits * NoVisitsS) + (CostUVisit * NoVisitsU) + CostSupp$$

, where NoVisitsD, NoVisitsS, NoVisitsU= number of visits for attending DOTs, scheduled and unscheduled visits respectively.

All participants, conditional on survival to week 76, will be included in the primary analysis. In the secondary analysis, all modified intention to treat participants will be included, treating missing answers as missing data and handled as explained in the missing data section below.

All costs will be collected in the local currency and converted to USD using the exchange rate reported by OANDA[8] at the time of the analysis. All costs will be inflated to 2021 prices.

The analysis will be performed in Stata (Stata Corp., USA) and for each cost category, descriptive statistics (mean, median, standard error and interquartile range) will be presented.

Quality assurance exercises will be carried out regularly during data collection by the central Health Economics team, to assess the logic and credibility of responses. Feedback will be provided to data collection staff on any issues raised from the exercise, so that they could correct and improve their guidance to participants during data collection.

HRQoL measurement

For the primary outcome calculations, patient health states will be measured prospectively using the EQ-5D-5L[9] every 12 weeks from randomisation until week 76. The responses to the questionnaire will be converted into health utility scores using the most appropriate tariff for each country, selected based on geographical proximity and economic context. Currently, the tariffs that we propose to use are from Indonesia (for India), Ethiopia (for Ethiopia and Uganda) and Poland (for Moldova). We will use updated value sets if these become available before the analysis stage. The value sets will be used to calculate the HRQoL for each patient at each interview point. Observations for each patient will be

For peer review only

combined to calculate a QALY score for each arm using the ‘area under the curve’ linear method, using the formula below:

$$\text{QALY} = \Sigma\,[(U_i + U_{i+1})/2] \times (t_{i+1} - t_i).$$

Where U= utility value and t= time between interviews.

QALY calculations will also account for mortality during the follow-up period, by assigning 0 QALYs from time of death until the end of follow up.

The health system costs will be calculated on a per patient basis and together with the QALY outcome will be used to calculate the Incremental cost-effectiveness ratio (ICER) of regimen C to regimen B, using the formula below:

$$ICER = \frac{(Cost_{RegimenC} - Cost_{RegimenB})}{(Mean\ QALY_{RegimenC} - Mean\ QALY_{RegimenB})}$$

Cost-effectiveness acceptability curves (CEAC) will be constructed to compare the regimens’ probabilities of being cost-effective against a set of pre-set threshold values.

Secondary objectives

Secondary objectives will consider the primary clinical outcome in the clinical trial. This is a favourable outcome, where a participant had their last two culture results, taken on separate visits but no more than six weeks earlier than week 76, negative or an unfavourable outcome.

For the societal perspective analyses, patient data collected will be added to the health system costs to calculate the societal costs.

Sub-group analyses

We will present data disaggregated by age, sex, HIV status, site and other variables may be presented where they will be identified in the study as potentially relevant.

Missing data

The nature and pattern of missing data will be analysed. If necessary, multiple imputation techniques[10] will be used to address the missing data in the base case, by using relevant baseline variables. This method is recommended for economic evaluations alongside clinical trials[11]. Other methods such as complete case analysis, average imputation, lowest and highest point imputation and listwise deletion will be tested in the sensitivity analysis- see table 2.

Statistical analysis

We will present our results in terms of precision, i.e. how close the data are expected to be to the true population value. 95% CI ranges will be constructed and presented such that there is a 95% probability that the results will contain the true population parameter[12].

Sensitivity analyses

For peer review only

Sensitivity analyses will be used to test the robustness of the results. Planned sensitivity analyses can be seen in the table below, however, any other things that become important will also be tested.

Parameters	Rationale/Method
Complete-case analysis, Average imputation, Lowest and Highest point imputation	If the level of missing observations for costs and HRQoL is higher than 10%, the MI technique is more prone to bias. Data sets will be analysed to assess whether the results indicate similar conclusions
Data collected retrospectively in India and Uganda	As some data have been collected retrospectively during the trial due to logistics issues, two data sets, one including the retrospectively collected data (where recall bias might have occurred) and one excluding it will be analysed to assess whether the results indicate similar conclusions
On the most important cost drivers	Unit costs will vary across different sites in the same country. Therefore, deterministic sensitivity analysis will be conducted to assess whether the results change as unit costs of the most important cost drivers are varied within plausible ranges.
Parameter uncertainty	Probabilistic sensitivity analysis to explore uncertainties surrounding key parameters; 1000 simulations will be run, and results presented as mean costs and QALYs.
Inpatient stay	Since 2011 WHO recommends outpatient models of care for MDR-TB patients. The analysis will be re-run excluding inpatient stay costs

Table 4

A nonparametric bootstrapping approach will be used to determine the level of sampling uncertainty surrounding the mean ICER by generating 10,000 estimates of incremental costs and outcomes. These will be presented on a cost-effectiveness plane. Net Monetary Benefit (NMB) will be calculated for each bootstrap estimate for a range of cost-effectiveness thresholds as follows:

$$NMB = (\lambda * \Delta \text{QALYs}) - \Delta \text{Costs}$$

, where λ represents the cost-effectiveness threshold. This will be calculated as 1 to 3 times GDP per capita, and other thresholds from country guidance or the literature. The regimen with NMB>0 or with the highest NMB should be adopted. Mean NMB will be reported with 95% bootstrap confidence intervals and z-test conducted.

COVID-19 impact

Also, the COVID-19 outbreak started during the trial. It is expected that the lockdown measures taken in most countries will affect the socioeconomic status of the patients and their quality of life, independent of their MDR-TB or MDR-TB treatment. There are a few measures that will be taken to record this. A COVID19 diary, containing information about

For peer review only

the lockdown restrictions will be completed by each site. Also, an additional questionnaire has been developed to further explore some of the answers regarding their income, spending and health-related quality of life.

As data collection started in 2015, before the outbreak, the lockdown imposed will be modelled as an independent explanatory variable for parameters such as quality of life, working hours and supplements spending during intensive, continuation and post-treatment phase. If the variable turns out to be significant, we will use it to adjust values reported post-pandemic, using model predictions of what would have been reported if the pandemic hadn't happened.

Additional changes to the protocol as a result of COVID19 may be implemented as needed.

Discussion

STREAM will be the first study to estimate the costs incurred by both patients undergoing MDR-TB treatment and the health care system within a Phase III RCT.

The detailed costing and analysis in four different settings will provide insights into the timing and drivers of the cost saving or dissaving of implementing an all oral regimen, providing the data for targeted interventions if needed.

The study will have certain limitations. The EQ-5D-5L is not a condition-specific measure, and so may miss differences in symptoms that are important to participants. Also, our method assumes a linear relationship between values at different time points, however, this might not be accurate. It was considered not feasible to ask participants to complete the EQ-5D-5L questionnaire at a more frequent interval, i.e. each DOT visit.

The trial setting also means that the experience of participants might be different from routine practice, in ways that could influence costs, such as the frequency of visits and their location and the provision of support (e.g. transport vouchers, food vouchers).

Word count: 2903

Funding

This article is made possible by the generous support of the American people through the United States Agency for International Development (USAID). The contents are the responsibility of the authors and do not necessarily reflect the views of USAID or the United States Government. Stage 2 is funded by the United States Agency for International Development (USAID) and Janssen Pharmaceuticals, with additional funding from the UK Medical Research Council and the UK Department for International Development (DFID). It is sponsored by Vital Strategies, Inc. (an affiliate of The International Union Against Tuberculosis and Lung Disease).

Conflict of interest statement

The authors and collaborating authors have no conflicts of interest to declare.

For peer review only

STREAM Study Health Economic Evaluation Collaborators

Mamo Girma, Vanita Patel, Makwana Mukesh, Malaisamy Muniyandi, Shravan Kumar, Sangeetha Subramani, Saleem Ahmad, Jasper Nidoi, Irina Pirlog, Mariana Macarie, I.D. Rusen, Gay Bronson, Meera Gurumurthy, Karen Sanders, Sarah Meredith, Andrew Nunn, Ben Spittle, Wendy Dodds, Robyn Henry-Cockles, Rachel Bennett, Elisa Giallongo, Danni Maas, Rachel Bennett, Ruth Goodall, Saiam Ahmed, Claire Cook, Katharine Bellenger, Gopalan Narendran, Bruce Kirenga, Elena Tudor, Rajesh Solanki, Daniel Kokeb, Adamu Bayissa

REFERENCES

1. World Health Organization. (2018). Global tuberculosis report 2018.
2. World Health Organization. (2014). The End TB Strategy: Global strategy targets for tuberculosis prevention, care and control after 2015.
3. World Health Organization. (2019). Rapid communication: key changes to treatment of drug-resistant tuberculosis.
4. World Health Organization. (2018). Rapid communications: key changes to treatment of multidrug- and rifampicin-resistant tuberculosis (MDR/RR-TB).
5. The STREAM protocol v8.0
6. Madan, Jason J, Rosu, Laura, Tefera, Mamo Girma, van Rensburg, Craig, Evans, Denise. et al. (2020). Economic evaluation of short treatment for multidrug-resistant tuberculosis, Ethiopia and South Africa: the STREAM trial. Bulletin of the World Health Organization, 98 (5), 306 - 314. http://dx.doi.org/10.2471/BLT.19.243584
7. The Tool to Estimate Patients' Cost. (2008). TB|CTA (http://www.stoptb.org/wg/dots_expansion/tbandpoverty/assets/documents/Tool%20to%20estimate%20Patients'%20Costs.pdf)
8. OANDA.com. Currency Converter (https://www1.oanda.com/currency/converter/)
9. EuroQol. (https://euroqol.org/eq-5d-instruments/eq-5d-5l-about/
10. Royston P. Multiple imputations of missing values. (2004). The Stata Journal 2004, 3:227-241.

For peer review only

11. ISPOR. Good Practices for Outcomes Research (https://www.ispor.org/heor-resources/good-practices-for-outcomes-research)

12. Andrew A. Anderson (2019) Assessing Statistical Results: Magnitude, Precision, and Model Uncertainty, The American Statistician, 73:sup1, 118-121, DOI: 10.1080/00031305.2018.1537889X. http://www.stoptb.org/wg/dots_expansion/tbandpoverty/assets/documents/Tool%20to%20estimate%20Patients'%20Costs.pdf

Annex1

CHEERS Checklist

Section	Item No	Recommendation	Reported on page No/line No
**Title and Abstract**			
**Title**	1	Identify the study as an economic evaluation or use more specific terms such as "cost-effectiveness analysis", and describe the interventions compared.	Title is: Economic evaluation of a short, all-oral bedaquiline-containing regimen for the treatment of rifampicin-resistant tuberculosis from the STREAM trial
**Abstract**	2	Provide a structured summary of objectives, perspective, setting, methods (including study design and inputs), results (including base case and uncertainty analyses), and conclusions.	N/A as it is an analysis plan
**Introduction**			
**Background and objectives**	3	Provide an explicit statement of the broader context for the study.<br><br>Present the study question and its relevance for health policy or practice decisions.	Covered in Background and Objectives sections
**Methods**			
**Target population and subgroups**	4	Describe characteristics of the base case population and subgroups analyzed, including why they were chosen.	Covered in the Methods and Analysis section
**Setting and location**	5	State relevant aspects of the system(s) in which the decision(s) need(s) to be made.	Covered in the Methods and Analysis section
**Study perspective**	6	Describe the perspective of the study and relate this to the costs being evaluated.	Covered in the Methods and Analysis section
**Comparators**	7	Describe the interventions or strategies being compared and state why they were chosen.	Covered in the Methods and Analysis section
**Time horizon**	8	State the time horizon(s) over which costs and consequences are being evaluated and say why appropriate.	Covered in the Methods and Analysis section
**Discount rate**	9	Report the choice of discount rate(s) used for costs and outcomes and say why appropriate.	In the Methods section, Health

For peer review only

			system resource use and cost sub-heading
**Choice of health outcomes**	10	Describe what outcomes were used as the measure(s) of benefit in the evaluation and their relevance for the type of analysis performed.	Covered in the Methods and Analysis section
**Measurement of effectiveness**	11a	*Single study-based estimates*: Describe fully the design features of the single effectiveness study and why the single study was a sufficient source of clinical effectiveness data.	Reference to the clinical paper; Covered in the Methods and Analysis section
	11b	*Synthesis-based estimates*: Describe fully the methods used for identification of included studies and synthesis of clinical effectiveness data.	N/A
**Measurement and valuation of preference-based outcomes**	12	If applicable, describe the population and methods used to elicit preferences for outcomes.	N/A
**Estimating resources and costs**	13a	*Single study-based economic evaluation*: Describe approaches used to estimate resource use associated with the alternative interventions. Describe primary or secondary research methods for valuing each resource item in terms of its unit cost. Describe any adjustments made to approximate to opportunity costs.	Fully described in Methods and Analysis section
	13b	*Model-based economic evaluation*: Describe approaches and data sources used to estimate resource use associated with model health states. Describe primary or secondary research methods for valuing each resource item in terms of its unit cost. Describe any adjustments made to approximate to opportunity costs.	N/A
**Currency, price date, and conversion**	14	Report the dates of the estimated resource quantities and unit costs. Describe methods for adjusting estimated unit costs to the year of reported costs if necessary. Describe methods for converting costs into a common currency base and the exchange rate.	Dates of the estimated resource quantities and unit costs not reported as this is a protocol. Methods for adjusting the unit costs and converting costs into a common currency are covered in the Methods and Analysis section, after the Patient costs sub-heading.
**Choice of model**	15	Describe and give reasons for the specific type of decision analytical model used. Providing a figure to show model structure is strongly recommended.	N/A as not a model-based evaluation
**Assumptions**	16	Describe all structural or other assumptions underpinning the decision-analytical model.	N/A as not a model-based evaluation
**Analytical methods**	17	Describe all analytical methods supporting the evaluation. This could include methods for dealing with skewed, missing, or censored data; extrapolation methods; methods for pooling data; approaches to validate or make adjustments (such as half cycle corrections) to a model; and methods for handling population heterogeneity and uncertainty.	Fully covered in the Methods and Analysis section, in the Missing data, Statistical analysis and Sensitivity analyses sub-sections.
**Results**			
**Study parameters**	18	Report the values, ranges, references, and, if used, probability distributions for all parameters. Report reasons or sources for distributions used to represent uncertainty where appropriate. Providing a table to show the input values is strongly recommended.	N/A as this is a study protocol, but these will be presented in the main paper as stated in this protocol
**Incremental costs and outcomes**	19	For each intervention, report mean values for the main categories of estimated costs and outcomes of interest, as well as mean differences between the comparator groups. If applicable, report incremental cost-effectiveness ratios.	N/A as this is a study protocol, but these will be presented in the main paper as stated in this protocol

For peer review only

**Characterizing uncertainty**	**20a**	*Single study-based economic evaluation*: Describe the effects of sampling uncertainty for the estimated incremental cost and incremental effectiveness parameters, together with the impact of methodological assumptions (such as discount rate, study perspective).	Methods and Analysis section of the protocol-Sensitivity analyses sub-heading.
	20b	*Model-based economic evaluation*: Describe the effects on the results of uncertainty for all input parameters, and uncertainty related to the structure of the model and assumptions.	N/A
**Characterizing heterogeneity**	21	If applicable, report differences in costs, outcomes, or cost-effectiveness that can be explained by variations between subgroups of patients with different baseline characteristics or other observed variability in effects that are not reducible by more information.	Costs and outcomes will be presented separately for each country
**Discussion**			
**Study findings, limitations, generalizability, and current knowledge**	22	Summarise key study findings and describe how they support the conclusions reached. Discuss limitations and the generalizability of the findings and how the findings fit with current knowledge.	Discussion about the strengths and limitations in the Discussion section; the key findings and their generalizability will be presented in the paper.
**Other**			
**Source of funding**	23	Describe how the study was funded and the role of the funder in the identification, design, conduct, and reporting of the analysis. Describe other non-monetary sources of support.	Acknowledgements
**Conflicts of interest**	24	Describe any potential for conflict of interest of study contributors in accordance with journal policy. In the absence of a journal policy, we recommend authors comply with International Committee of Medical Journal Editors recommendations.	Acknowledgements

Annex2

Value sets to be used

For peer review only

Table 3 Estimation results for C-TTO model, DCE rescaled model, and hybrid model

Independent variables of the model	C-TTO Tobit model censored at −1			DCE conditional logistic model rescaled			Hybrid model censored C-TTO values at −1 (final value set)		
	Coeff.	(SE)	*p* value	Coeff.	(SE)	*p* value	Coeff.	(SE)	*p* value
Mobility (MO)									
No problems to slight problems	0.088	(0.015)	0.000	0.139	(0.015)	0.000	0.119	(0.008)	0.000
Slight problems to moderate problems	0.086	(0.017)	0.000	0.080	(0.017)	0.000	0.073	(0.011)	0.000
Moderate problems to severe problems	0.250	(0.019)	0.000	0.196	(0.016)	0.000	0.218	(0.013)	0.000
Severe problems to unable	0.170	(0.018)	0.000	0.219	(0.018)	0.000	0.203	(0.012)	0.000
Self-care (SC)									
No problems to slight problems	0.085	(0.014)	0.000	0.101	(0.016)	0.000	0.101	(0.007)	0.000
Slight problems to moderate problems	0.056	(0.018)	0.002	0.038	(0.018)	0.032	0.039	(0.010)	0.000
Moderate problems to severe problems	0.128	(0.018)	0.000	0.085	(0.019)	0.000	0.108	(0.013)	0.000
Severe problems to unable	0.035	(0.016)	0.030	0.097	(0.017)	0.000	0.068	(0.012)	0.000
Usual activities (UA)									
No problems to slight problems	0.071	(0.015)	0.000	0.092	(0.016)	0.000	0.090	(0.006)	0.000
Slight problems to moderate problems	0.106	(0.017)	0.000	0.051	(0.017)	0.003	0.066	(0.011)	0.000
Moderate problems to severe problems	0.137	(0.019)	0.000	0.154	(0.017)	0.000	0.145	(0.013)	0.000
Severe problems to unable	0.061	(0.018)	0.001	0.091	(0.017)	0.000	0.084	(0.013)	0.000
Pain/discomfort (PD)									
No problems to slight problems	0.089	(0.013)	0.000	0.081	(0.016)	0.000	0.086	(0.006)	0.000
Slight problems to moderate problems	0.007	(0.019)	0.721	0.012	(0.018)	0.513	0.009	(0.011)	0.395
Moderate problems to severe problems	0.135	(0.018)	0.000	0.085	(0.017)	0.000	0.103	(0.013)	0.000
Severe problems to extreme problems	0.024	(0.019)	0.211	0.053	(0.018)	0.003	0.048	(0.013)	0.000
Anxiety/depression (AD)									
No problems to slight problems	0.079	(0.014)	0.000	0.050	(0.017)	0.003	0.079	(0.006)	0.000
Slight problems to moderate problems	0.055	(0.018)	0.002	0.061	(0.017)	0.000	0.055	(0.011)	0.000
Moderate problems to severe problems	0.086	(0.017)	0.000	0.114	(0.018)	0.000	0.093	(0.012)	0.000
Severe problems to extreme problems	0.062	(0.016)	0.000	0.085	(0.018)	0.000	0.078	(0.012)	0.000
Log likelihood	−6189.97			−3958.62			−9325.84		
AIC	12,421.93			7957.24			18,735.69		
BIC	12,572.19			8109.23			19,060.41		

Value set to be used for India. Purba FD, Hunfeld JAM, Iskandarsyah A, et al. The Indonesian EQ-5D-5L Value Set. Pharmacoeconomics. 2017;35(11):1153-1165. doi:10.1007/s40273-017-0538-9

Table 3. Estimation results for *c*-TTO model, DCE rescaled model, and hybrid model in incremental dummies.*

Independent variables of the model	c-TTO ordinary least squares model			DCE conditional logistic model rescaled			Hybrid model censored c-TTO values at −1 (final value set)		
	Coefficient	SE	*P* value	Coefficient	SE	*P* value	Coefficient	SE	*P* value
Mobility (MO)									
MO2	0.0047	0.014	.729	0.4780	0.061	.000	0.0337	0.005	.000
MO3	0.0166	0.015	.262	0.1138	0.071	.110	0.0307	0.009	.000
MO4	0.1748	0.016	.000	0.9810	0.070	.000	0.1632	0.010	.000
MO5	0.1038	0.016	.000	0.7434	0.074	.000	0.1322	0.010	.000
Self-care (SC)									
SC2	0.0036	0.013	.785	0.2044	0.067	.002	0.0235	0.005	.000
SC3	0.0494	0.016	.002	−0.0024	0.074	.974	0.0160	0.008	.042
SC4	0.1189	0.015	.000	0.6849	0.078	.000	0.1024	0.009	.000
SC5	0.0826	0.013	.000	0.4234	0.073	.000	0.0804	0.009	.000
Usual activities (UA)									
UA2	0.0188	0.014	.176	0.3470	0.063	.000	0.0323	0.005	.000
UA3	0.0441	0.014	.002	−0.0391	0.071	.579	0.0160	0.008	.042
UA4	0.1299	0.016	.000	0.5818	0.071	.000	0.1091	0.009	.000
UA5	0.0936	0.015	.000	0.6079	0.076	.000	0.1147	0.010	.000
Pain/discomfort (PD)									
PD2	0.0140	0.013	.266	0.4499	0.067	.000	0.0361	0.004	.000
PD3	0.0161	0.017	.331	0.1090	0.073	.136	0.0155	0.008	.061
PD4	0.2452	0.015	.000	1.1358	0.077	.000	0.2187	0.010	.000
PD5	0.1421	0.016	.000	0.5689	0.076	.000	0.1361	0.011	.000
Anxiety/depression (AD)									
AD2	0.0111	0.014	.428	0.2718	0.070	.000	0.0259	0.004	.000
AD3	0.0381	0.015	.012	0.3516	0.072	.000	0.0589	0.008	.000
AD4	0.2322	0.015	.000	1.1803	0.079	.000	0.2139	0.009	.000
AD5	0.1414	0.013	.000	0.8320	0.078	.000	0.1591	0.010	.000
Akaike information criterion	10 587.06			6498.30		14 002.09			
Bayesian information criterion	10 739.33			6650.17		14 336.81			
Order of importance									

Value set to be used for Uganda and Ethiopia. Welie AG, Gebretekle GB, Stolk E, Mukuria C, Krahn MD, Enquoselassie F, Fenta TG. Valuing health state: an EQ-5D-5L value set for Ethiopians. Value Health Reg Issues. 2019;22:7–14

Table 2 Modelling results

	Model 1 panel, random effects	Model 2 Bayesian	Model 3 M2 + random parameters	Model 4 M3 + error scaling with *t*-Student	Model 5 M4 + religion scaling	Final model M5 + DCE, censoring
Const.	0.005 (−0.010; 0.019)	Not used	Not used	Not used	Not used	Not used
MO2	0.021 (0.002; 0.039)	0.023 (0.001; 0.044)	0.058 (0.013; 0.073)	0.017 (0.014; 0.022)	0.019 (0.014; 0.023)	0.025 (0.020; 0.029)
MO3	0.012 (−0.007; 0.031)	0.016 (0.000; 0.036)	0.077 (0.021; 0.094)	0.015 (0.005; 0.026)	0.016 (0.005; 0.028)	0.034 (0.026; 0.042)
MO4	0.098 (0.077; 0.118)	0.101 (0.074; 0.129)	0.159 (0.071; 0.181)	0.101 (0.085; 0.116)	0.107 (0.090; 0.124)	0.126 (0.113; 0.141)
**MO5**	**0.262 (0.238; 0.285)**	**0.263 (0.239; 0.289)**	**0.303 (0.271; 0.330)**	**0.251 (0.228; 0.274)**	**0.267 (0.242; 0.293)**	**0.314 (0.286; 0.342)**
SC2	0.030 (0.014; 0.046)	0.037 (0.015; 0.059)	0.015 (0.003; 0.087)	0.029 (0.024; 0.034)	0.031 (0.026; 0.036)	0.031 (0.027; 0.036)
SC3	0.038 (0.017; 0.059)	0.042 (0.014; 0.071)	0.005 (0.000; 0.119)	0.037 (0.028; 0.047)	0.040 (0.029; 0.050)	0.047 (0.040; 0.055)
SC4	0.122 (0.098; 0.146)	0.116 (0.089; 0.143)	0.042 (0.027; 0.180)	0.108 (0.094; 0.123)	0.115 (0.099; 0.131)	0.111 (0.099; 0.123)
**SC5**	**0.276 (0.254; 0.298)**	**0.269 (0.244; 0.295)**	**0.242 (0.193; 0.268)**	**0.258 (0.237; 0.282)**	**0.273 (0.249; 0.299)**	**0.264 (0.243; 0.286)**
UA2	0.031 (0.014; 0.048)	0.034 (0.011; 0.058)	0.002 (0.000; 0.007)	0.033 (0.026; 0.039)	0.034 (0.028; 0.042)	0.023 (0.019; 0.027)
UA3	0.032 (0.009; 0.054)	0.041 (0.015; 0.067)	0.005 (0.000; 0.014)	0.050 (0.040; 0.060)	0.053 (0.043; 0.063)	0.040 (0.032; 0.048)
UA4	0.092 (0.070; 0.115)	0.088 (0.062; 0.115)	0.024 (0.010; 0.038)	0.104 (0.091; 0.117)	0.110 (0.095; 0.125)	0.097 (0.087; 0.107)
**UA5**	**0.186 (0.167; 0.206)**	**0.183 (0.157; 0.209)**	**0.180 (0.161; 0.201)**	**0.180 (0.161; 0.200)**	**0.190 (0.169; 0.212)**	**0.205 (0.188; 0.224)**
PD2	0.028 (0.012; 0.044)	0.033 (0.012; 0.054)	0.041 (0.028; 0.054)	0.025 (0.021; 0.028)	0.026 (0.022; 0.030)	0.030 (0.026; 0.034)
PD3	0.034 (0.014; 0.053)	0.035 (0.007; 0.063)	0.053 (0.036; 0.071)	0.030 (0.022; 0.039)	0.032 (0.022; 0.041)	0.050 (0.043; 0.058)
PD4	0.229 (0.208; 0.251)	0.228 (0.204; 0.254)	0.253 (0.224; 0.276)	0.223 (0.208; 0.239)	0.235 (0.217; 0.253)	0.261 (0.244; 0.280)
**PD5**	**0.467 (0.440; 0.494)**	**0.473 (0.446; 0.499)**	**0.490 (0.464; 0.518)**	**0.492 (0.463; 0.520)**	**0.519 (0.485; 0.555)**	**0.575 (0.538; 0.613)**
AD2	0.024 (0.006; 0.041)	0.032 (0.010; 0.054)	0.049 (0.015; 0.061)	0.019 (0.016; 0.023)	0.020 (0.017; 0.024)	0.018 (0.015; 0.021)
AD3	0.034 (0.011; 0.056)	0.033 (0.006; 0.058)	0.085 (0.038; 0.101)	0.037 (0.026; 0.049)	0.039 (0.027; 0.052)	0.029 (0.022; 0.037)
AD4	0.114 (0.094; 0.135)	0.114 (0.088; 0.139)	0.160 (0.116; 0.181)	0.119 (0.106; 0.132)	0.126 (0.113; 0.142)	0.108 (0.097; 0.119)
**AD5**	**0.224 (0.203; 0.244)**	**0.226 (0.201; 0.251)**	**0.176 (0.153; 0.231)**	**0.211 (0.194; 0.229)**	**0.223 (0.204; 0.243)**	**0.232 (0.213; 0.252)**
Deviance	61.2% (R^2 used instead)	11,866	−777	−13,781	−13,780	−9215
DIC		11,886	2597	−9704	−9704	−9215[a]
PSRF	n.a.	All <1.01	Maximum = 15	All <1.01	All <1.01	All <1.01

Value set to be used for Moldova. Golicki, D., Jakubczyk, M., Graczyk, K. et al. Valuation of EQ-5D-5L Health States in Poland: the First EQ-VT-Based Study in Central and Eastern Europe. PharmacoEconomics 37, 1165–1176 (2019). https://doi.org/10.1007/s40273-019-00811-7

For peer review only

BMJ Open

Economic evaluation protocol of a short, all-oral bedaquiline-containing regimen for the treatment of rifampicin-resistant tuberculosis from the STREAM trial

Journal:	*BMJ Open*
Manuscript ID	bmjopen-2020-042390.R1
Article Type:	Protocol
Date Submitted by the Author:	11-Nov-2020
Complete List of Authors:	Rosu, Laura; Liverpool School of Tropical Medicine, Centre for Applied Health Research and Delivery & Department of Clinical Sciences<br>Madan, Jason; University of Warwick, Warwick Medical School<br>Worrall, E; Liverpool School of Tropical Medicine,<br>Tomeny, Ewan; Liverpool School of Tropical Medicine, Centre for Applied Health Research and Delivery & Department of Clinical Sciences<br>Squire, Bertie; Liverpool school of tropical medicine, Centre for Applied Health Research and Delivery & Department of Clinical Sciences
**Primary Subject Heading**:	Health economics
Secondary Subject Heading:	Research methods, Respiratory medicine, Infectious diseases
Keywords:	Tuberculosis < INFECTIOUS DISEASES, Economics < TROPICAL MEDICINE, Health economics < HEALTH SERVICES ADMINISTRATION & MANAGEMENT

SCHOLARONE™
Manuscripts

I, the Submitting Author has the right to grant and does grant on behalf of all authors of the Work (as defined in the below author licence), an exclusive licence and/or a non-exclusive licence for contributions from authors who are: i) UK Crown employees; ii) where BMJ has agreed a CC-BY licence shall apply, and/or iii) in accordance with the terms applicable for US Federal Government officers or employees acting as part of their official duties; on a worldwide, perpetual, irrevocable, royalty-free basis to BMJ Publishing Group Ltd ("BMJ") its licensees and where the relevant Journal is co-owned by BMJ to the co-owners of the Journal, to publish the Work in this journal and any other BMJ products and to exploit all rights, as set out in our licence.

The Submitting Author accepts and understands that any supply made under these terms is made by BMJ to the Submitting Author unless you are acting as an employee on behalf of your employer or a postgraduate student of an affiliated institution which is paying any applicable article publishing charge ("APC") for Open Access articles. Where the Submitting Author wishes to make the Work available on an Open Access basis (and intends to pay the relevant APC), the terms of reuse of such Open Access shall be governed by a Creative Commons licence – details of these licences and which Creative Commons licence will apply to this Work are set out in our licence referred to above.

Other than as permitted in any relevant BMJ Author's Self Archiving Policies, I confirm this Work has not been accepted for publication elsewhere, is not being considered for publication elsewhere and does not duplicate material already published. I confirm all authors consent to publication of this Work and authorise the granting of this licence.

For peer review only

Economic evaluation protocol of a short, all-oral bedaquiline-containing regimen for the treatment of rifampicin-resistant tuberculosis from the STREAM trial

Laura Rosu[1], Jason J Madan[2], Eve Worrall[3], Ewan Tomeny[1], S Bertel Squire[1] and the STREAM Study Health Economic Evaluation Collaborators

[1] Centre for Applied Health Research and Delivery, Liverpool School of Tropical Medicine, Liverpool, UK

[2] Warwick Medical School, University of Warwick, Coventry, UK

[3] Vector Biology, Liverpool School of Tropical Medicine, Liverpool, UK

Corresponding author: Laura Rosu, laura.rosu@lstmed.ac.uk

Keywords: tuberculosis, multi-drug resistant TB, cost-effectiveness analysis; cost-utility analysis; economic evaluation; short multi-drug resistant treatment; bedaquiline TB treatment; applied health research

Abstract

Introduction

A December 2019 WHO rapid communication recommended the use of nine-month, all-oral regimens for treating multi-drug resistant tuberculosis (MDR-TB). Besides the clinical benefits, they are thought to be less costly than the injectable-containing regimens, for both the patient and the health system. STREAM is the first randomised controlled trial with an economic evaluation to compare all-oral and injectable-containing 9-11 month MDR-TB treatment regimens.

Methods and Analysis

Health system costs of delivering a nine-month, injectable containing regimen and a nine-month all-oral bedaquiline-containing regimen will be collected in Ethiopia, India, Moldova, and Uganda, using 'bottom-up' and 'top-down' costing approaches. Patient-costs will be collected using questionnaires that have been developed based on the STOP-TB questionnaire. The primary objective of the study is to estimate the cost-utility of the two regimens, from a health system perspective. Secondary objectives include estimating the cost-utility from a societal perspective as well as evaluating the cost-effectiveness of the regimens, using both health system and societal perspectives. The effect measure for the

For peer review only - http://bmjopen.bmj.com/site/about/guidelines.xhtml

cost-utility analysis will be the QALY, while the effect measure for the cost-effectiveness analysis will be the efficacy outcome from the clinical trial.

Ethics and dissemination

The study has been evaluated and approved by the Ethics Advisory Group of the *International Union Against Tuberculosis and Lung Disease* and also approved by ethics committees in all participating countries. All participants have provided written informed consent. The results of the economic evaluation will be published in a peer-reviewed journal.

Trial registration number: ISRCTN18148631

For peer review only

Strengths and limitations of this study

- The economic evaluation of STREAM will be the first study to estimate the costs incurred by both patients undergoing MDR-TB treatment and the health care system within a Phase III RCT.
- The detailed costing and analysis in four different settings will provide valuable insights into the timings and drivers of the costs associated with implementation of a nine month all-oral bedaquiline-containing regimen. The study will generate important evidence needed for future policy decisions, and the shaping of targeted interventions.
- The trial setting means that additional research costs (e.g. costs for collecting pharmacokinetic samples, social support costs paid for by the study) that would not be incurred in a routine setting will be incurred. These research costs will be separated out and eliminated from the costing analysis. Additionally, the experience of participants and delivery of health services (e.g. frequency of visits) will in places, inevitably deviate from routine practice, with implications for patient and health system costs. Though we will attempt to adjust for these differences in analysis, guaranteeing no interference may not be possible.

Background

The STREAM trial is a phase III non-inferiority RCT to test the efficacy, safety and economic impact of shortened multi-drug resistant tuberculosis (MDR-TB) treatment regimens. MDR-TB is a form of tuberculosis (TB) caused by bacteria that cannot be treated with two of the most powerful, first-line anti-TB drugs, isoniazid and rifampicin. Globally, in 2017, there were a little over half a million people with TB resistant to rifampicin, and out of these 82% had MDR-TB[1].

For peer review only - http://bmjopen.bmj.com/site/about/guidelines.xhtml

The World Health Organization's (WHO) End TB Strategy is among the health targets of the Sustainable Development Goals. It was adopted by the World Health Assembly in 2014 with the aim of reducing TB deaths by 90% and new cases by 80% between 2015 and 2030, as well as reducing to zero the number of households incurring catastrophic costs due to TB by 2020. Currently, global TB incidence is falling at 2% per year, which is insufficient to reach the 2020 milestone[2]. This means that new ways of addressing the disease must be found to meet these targets. Careful evaluation of alternative treatment strategies is vital to ensure the most effective and feasible approaches are implemented.

The December 2019 WHO rapid communication recommends the use of shorter, all-oral, bedaquiline-containing regimens for MDR-TB patients[3]. It seems that all-oral regimens, as opposed to those containing injectables, are becoming the preferred option for treatment of MDR-TB as data from the South African TB programme had suggested them to improve patient outcomes. Replacing the injectable with bedaquiline resulted in better treatment success and better adherence[3]. Beside the clinical benefits, it is also thought that the all-oral treatment leads to lower costs from a health system and patient perspective[4]. It is therefore crucial to test these hypotheses via a randomised controlled trial (RCT) in multiple settings. Furthermore, to date, no phase III trial has included an economic analysis of the nine-month bedaquiline-containing regimen , making it difficult for policymakers to assess the economic and financial impact. STREAM is the first randomised phase III trial to include such an analysis, to compare the all-oral, bedaquiline-containing and injectable-containing 9-11 month MDR-TB treatment regimens.

Objectives

The questions that the economic evaluation is aiming to address include:

- What are the health system costs of treating MDR-TB patients using the following regimens: a 9-month injectable regimen; a 9-month all-oral bedaquiline containing regimen; and a 6-month injectable regimen?
- What costs do patients face during and after treatment?
- How does MDR-TB affect patients' socioeconomic situations?
- What financial coping mechanisms do patients employ?

The primary economic objective is to estimate the cost-utility of the two multi-drug resistant tuberculosis (MDR-TB) interventions, in each country, from a health system perspective. To achieve this, an economic evaluation of both the costs and consequences associated with each intervention will be conducted.

Secondary economic objectives include assessing the cost-utility of the regimens from a societal perspective and evaluating the cost-effectiveness of the regimens from both a health system and societal perspective.

For peer review only - http://bmjopen.bmj.com/site/about/guidelines.xhtml

The effect measure for the cost-utility analysis (CUA) will be the QALY, while the effect measure of the cost-effectiveness analysis (CEA) will be the efficacy outcome from the clinical trial i.e. favourable or unfavourable.

Methods and analysis

Randomised controlled trial design

Health economic data will be collected alongside the STREAM trial. Its protocol has been published elsewhere[5]. In brief, the STREAM study is an international, multi-centre, parallel-group RCT of patients with MDR-TB and patients with rifampicin resistant and isoniazid-sensitive TB. It will be assessed whether the proportion of participants on regimen C with a favourable efficacy outcome at week 76 is not less on that on regimen B i.e. C is non-inferior to B. Data will also be collected on regimen D for secondary comparisons. Treatments administered are outlined in Figure 1 and explained below:

Figure 1 Treatments outline. Regimen A was dropped of the trial.

Trial recruitment started in April 2016, across 13 sites in 7 countries (Table 1):

	Clinical trial sites	HE sites
**Mongolia**	National Center for Communicable Diseases, Ulaanbaatar	
**Ethiopia**	Armaeur Hansen Research Institute, Addis Ababa	x
	St. Peter's Hospital, Addis Ababa	x
**South Africa**	King Dinuzulu Hospital, Durban	
	Helen Joseph Hospital, Johannesburg	
	Empilweni TB Hospital, Port Elizabeth	
	Doris Goodwin, Pietermaritzburg	
**Moldova**	IMSP, Chiril Draganiuc, Chisinau	x
**Uganda**	Mulago Hospital, Kampala	x
**Georgia**	National Center for Tuberculosis and Lung Disease, Tbilisi	
**India**	B.J. Medical College, Ahmedabad	x
	National Institute for Research in Tuberculosis, Chennai	x
	Rajan Babu Institute for Pulmonary Medicine and Tuberculosis, Delhi	x

For peer review only - http://bmjopen.bmj.com/site/about/guidelines.xhtml

Table 1. STREAM trial sites

At the start of Stage 2 randomisation was to regimen A, regimen B, regimen C and regimen D, in a ratio of 1:2:2:2, done using a web-based system managed by MRC CTU. Version 8.0 of the protocol limits randomisation to arms B and C, so patients will no longer be randomised to regimen A and D and randomisation will be in a ratio of 1:1. At least 200 patients to each of regimen B and regimen C will be randomised, across all sites. This was determined based on the assumption that the proportion of patients with a favourable efficacy outcome at week76 is 80% for regimen B and 82% for regimen C. With a non-inferiority margin of 10% and a one-sided significance level of 2.5%, 180 evaluable patients will be required in each of the two regimens to demonstrate non-inferiority. If 10% of patients will be excluded from the primary efficacy analysis population, a total of 400 patients would be required in total for regimens B and C[5].

The health economic analysis will include participants of the clinical trial in the above-mentioned sites, who are over 18 years old and fulfil the inclusion/exclusion criteria as outlined in the trial protocol. All patients in the study will be followed up until week 132, with the primary analysis conducted on data collected up to week 76.

Patient data will be collected at 12-week intervals, during the patient assessment visits for the clinical trial, using a questionnaire developed based on the STOP-TB questionnaire, in all HE sites.

Health system cost data will be collected by the focal health economists in each country during the whole trial period.

The Consolidated Health Economic Evaluation Reporting Standards (CHEERS) checklist has been used as a guide to optimise the preparation and reporting of the methods used (Annex 1).

<u>Health system resource use and costs</u>

A mixture of top-down and bottom-up approaches will be used.

Data regarding staff time and staff activities involved in the management of MDR-TB treatment for each regimen will be collected by the focal health economists in each country using a standardised questionnaire developed by the health economic team, pilot tested in all HE sites and used in the first phase of the trial[6].

A full assessment of the health system costs of delivering the MDR-TB regimens, including tests performed, consumables used, inpatient stay costs, drugs administered, and overheads will be done in each country, for each arm. Any relevant resource events will also be included. These will be collected by the focal health economists in each country using hospitals' accounting records, clinical trial CRFs and STREAM protocol, and will be costed using local unit costs where possible. Where this will not be possible, STREAM or in-country private healthcare facilities costs will be used.

For peer review only - http://bmjopen.bmj.com/site/about/guidelines.xhtml

The costs associated with the diagnosis and management of serious adverse events (SAEs) caused by MDR-TB or its treatment will also be included. The costing will include all tests performed, examinations, investigations, in-patient stays and medication received, as well as staff costs. Data will be collected in an event costing tool developed in Microsoft Excel by the HE trial team and the main data source will be the clinical trial CRFs.

The total health system costs for each trial arm will be estimated by summing the costs of each resource used and presented by the following cost elements, by phase- see table 2 below:

Cost element	***Unit***	***Data sources***		***Method***
		Costs sources	***Quantity used per treatment phase (intensive, continuation and follow-up until week76)***	
**Inpatient stay**	Cost per day	Local hospitals' accounting records or local private facilities if not available	Actual number of inpatient stay days for all patients	Unit cost per day multiplied by the number of inpatient days for each patient
**Laboratory tests**	Cost per test	Local hospitals' laboratories or local private facilities if not available	Frequency from the STREAM trial protocol	Cost per test multiplied by the number of tests performed for each patient
**Medication**	Unit cost per tablet/dose	Local hospitals' pharmacies purchasing lists (alternative drug price lists if not available locally)	Dosages, treatment interruptions, etc. from the STREAM trial clinical CRFs	Unit cost per dose multiplied by the total number of doses for each patient
**Staff**	Cost per minute	Local pay scales	Time collected using staff questionnaire	Unit cost per minute multiplied by number of minutes in a visit multiplied by number of total visits
**Social support**	Cost per week	TB national programme	TB national programme	Cost per week times number of weeks the patient is eligible for social support
**Consumables**	Per patient per visit	Local hospitals' pharmacies purchasing lists or local private pharmacies	Quantity of each unit collected via direct observation and staff questionnaire	Unit cost per patient per visit multiplied by the number of visits.
**Serious adverse events**	Per patient per SAE	A combination of all the above	A combination of all the above	Unit costs of: consumables, lab tests, medication, staff will

For peer review only - http://bmjopen.bmj.com/site/about/guidelines.xhtml

				multiplied by the quantity of each to calculate the cost of managing each SAE
**Overheads**	Overhead costs per patient per day	As reported by the local hospitals accounting records	As reported by the local hospitals. Number of patients in the TB unit will be used as a proxy.	Total overhead costs will be calculated for the TB unit over a year, then divided by the number of TB patients in a year

Table 2. Health system costs sources and calculation methods

Capital costs extending beyond 1 year (e.g. equipment) will be annualised over their expected lifespan assuming a discount rate of 3%.

Research costs such as costs related to the pharmacokinetics study will not be collected or included in this economic evaluation. The health system costing will be done in close collaboration with the central health economic team to make sure it is sensible and evaluated with the support of a team of clinicians involved in the clinical trial. If deemed appropriate, other research costs that do not reflect usual practice will be excluded.

Patient costs

Patient costs will be collected by administering questionnaires that have been developed based on the STOP-TB questionnaire[7]. Data will be collected in two stages. First, a baseline questionnaire will capture socio-economic data of each patient before they start treatment. Then, a follow-up questionnaire capturing any changes to the socio-economic data and a patient treatment cost questionnaire will be administered every 12 weeks.

The patient costs to be collected are presented in Table 3:

**Cost type**	**Data collection method**	**Analysis**
Cost of attending DOTs (CostDots)	Through patient CRFs (transport and food costs data)	For each cost type category, data will be aggregated for each site and arm, to estimate the mean direct cost per visit
Costs of attending injection DOTs (CostDots)	Through patient CRFs (transport and food costs data)	
Patient cost for attending scheduled patient assessment visits (CostSVisits)	Through patient CRFs (transport and food costs data)	
Patient costs for attending unscheduled patient assessment visits (CostUVisits)	Through patient CRFs (transport and food costs data)	

For peer review only - http://bmjopen.bmj.com/site/about/guidelines.xhtml

Food supplements (CostSupp)	Through patient CRFs	Mean spend for each time point to be calculated and presented as the cumulative difference in food purchases between arms
Income loss during and after treatment	Reported by patients if willing to reveal their income at each time point; if not, working hours reported to be used as a proxy	If patients are unwilling to reveal their income, average salary values from the specific areas in each country will be used. The total lost hours will be multiplied with the hourly average wage. Total income loss during treatment and follow-up will be calculated

Table 3. Patient cost data collection method and analysis plan

The total direct cost per participant receiving MDR-TB treatment will be calculated as follows:

$$Total\ direct\ cost = (CostDots * NoVisitsD) + (CostSVisits * NoVisitsS) + (CostUVisit * NoVisitsU) + CostSupp$$

, where NoVisitsD, NoVisitsS, NoVisitsU= number of visits for attending DOTs, scheduled and unscheduled visits respectively.

Usually, TB patients are accompanied by a guardian to the direct observed treatment (DOT) and/or assessment visits. The guardians' direct costs (transport, food and accommodation costs) for each patient and for each visit will be included in the patient costs analysis. Patients who indicate they had a 'guardian' during treatment will be asked whether this guardian lost an income when accompanying them; their lost time will be assumed to equal the patient's and valued at the national minimum wage.

All participants, conditional on survival to week 76, will be included in the primary analysis. In the secondary analysis, all modified intention to treat participants will be included, treating missing answers as missing data and handled as explained in the missing data section below.

All costs will be collected in the local currency and converted to USD using the exchange rate reported by OANDA[8] at the time of the analysis. All costs will be inflated to 2021 prices.

Due to logistics issues, data collection for the health economic component was delayed at two Indian sites, Ahmedabad and Chennai, and the Ugandan site, so baseline and week 12 patient data will be collected at the week 24 or week 36 visit for the first patients enrolled into the trial. This will be subject to sensitivity analysis. All interviews after week36 will be conducted as scheduled, during the patient assessment visits.

The analysis will be performed in Stata (Stata Corp., USA) and for each cost category, descriptive statistics (mean, median, standard error and interquartile range) will be presented.

Quality assurance exercises will be carried out regularly during data collection by the central Health Economics team, to assess the logic and credibility of responses. Feedback will be

For peer review only

provided to data collection staff on any issues raised from the exercise, so that they could correct and improve their guidance to participants during data collection.

HRQoL measurement

For the primary outcome calculations, patient health states will be measured prospectively using the EQ-5D-5L[9] every 12 weeks from week 0 (i.e. baseline), before the patient takes the first drug, until week 76. The responses to the questionnaire will be converted into health utility scores using the most appropriate tariff for each country, selected based on geographical proximity and economic context. Currently, the tariffs that we propose to use are from Indonesia (for India), Ethiopia (for Ethiopia and Uganda) and Poland (for Moldova) and can be seen in annex 2. We will use updated value sets if these become available before the analysis stage. The value sets will be used to calculate the HRQoL for each patient at each interview point. Observations for each patient will be combined to calculate a QALY score for each arm using the 'area under the curve' linear method, using the formula below:

$$QALY = \sum \left[\frac{(U_i + U_{i+1})}{2} \right] \times (t_{i+1} - t_i)$$

Where U= utility value and t= time between interviews.

QALY calculations will also account for mortality during the follow-up period, by assigning 0 QALYs from time of death until the end of follow up.

The health system costs will be calculated on a per patient basis and together with the QALY outcome will be used to calculate the Incremental cost-effectiveness ratio (ICER) of regimen C to regimen B, using the formula below:

$$ICER = \frac{(Cost_{RegimenC} - Cost_{RegimenB})}{(Mean\ QALY_{RegimenC} - Mean\ QALY_{RegimenB})}$$

Cost-effectiveness acceptability curves (CEAC) will be constructed to compare the regimens' probabilities of being cost-effective against a set of pre-set threshold values, ranging from US$0 to US$100,000 and including some published estimates[10].

Secondary objectives

Secondary objectives will consider the primary clinical outcome in the clinical trial. This is a favourable outcome, where a participant had their last two culture results, taken on separate visits but no more than six weeks earlier than week 76, negative or an unfavourable outcome.

For the societal perspective analyses, direct patient costs data collected as explained above will be added to the health system costs to calculate the societal costs.

Sub-group analyses

We will present data disaggregated by age, sex, HIV status, site and other variables may be presented where they will be identified in the study as potentially relevant.

Missing data

For peer review only - http://bmjopen.bmj.com/site/about/guidelines.xhtml

The nature and pattern of missing data will be analysed. If necessary, multiple imputation techniques[11] will be used to address the missing data in the base case, by using relevant baseline variables. This method is recommended for economic evaluations alongside clinical trials[12]. Other methods such as complete case analysis, average imputation, lowest and highest point imputation and listwise deletion will be tested in the sensitivity analysis.

Statistical analysis

We will present our results in terms of precision, i.e. how close the data are expected to be to the true population value, presenting means and standard deviations of the results. 95% CI ranges will be constructed and presented such that there is a 95% probability that the results will contain the true population parameter[13].

Sensitivity analyses

Sensitivity analyses will be used to test the robustness of the results. Planned sensitivity analyses can be seen in table 4 below, however, any other things that become important will also be tested.

Parameters	Rationale/Method
Complete-case analysis, Average imputation, Lowest and Highest point imputation	If the level of missing observations for costs and HRQoL is higher than 10%, the MI technique is more prone to bias. Data sets will be analysed to assess whether the results indicate similar conclusions
Patient data collected retrospectively in India and Uganda	As some data have been collected retrospectively during the trial due to logistics issues, two data sets, one including the retrospectively collected data (where recall bias might have occurred) and one excluding it will be analysed to assess whether the results indicate similar conclusions
On the most important cost drivers	Unit costs will vary across different sites in the same country. Therefore, deterministic sensitivity analysis will be conducted to assess whether the results change as unit costs of the most important cost drivers are varied within plausible ranges.
Parameter uncertainty	Probabilistic sensitivity analysis to explore uncertainties surrounding key parameters; 1000 simulations will be run, and results presented as mean costs and QALYs.
Inpatient stay	Since 2011 WHO recommends outpatient models of care for MDR-TB patients. The analysis will be re-run excluding inpatient stay costs

Table 4. Planned sensitivity analyses

A nonparametric bootstrapping approach will be used to determine the level of sampling uncertainty surrounding the mean ICER by generating 10,000 estimates of incremental costs and outcomes. These will be presented on a cost-effectiveness plane. Confidence intervals

For peer review only - http://bmjopen.bmj.com/site/about/guidelines.xhtml

of the generated ICERs will then be calculated, in order to summarise the uncertainty due to sampling variations.

Net Monetary Benefit (NMB) will be calculated for each bootstrap estimate for a range of cost-effectiveness thresholds as follows:

$$NMB = (\lambda * \Delta\text{QALYs}) - \Delta\text{Costs}$$

, where λ represents the cost-effectiveness threshold. This will be calculated as 1 to 3 times GDP per capita, and other thresholds from country guidance or the literature. The regimen with NMB>0 or with the highest NMB should be adopted. Mean NMB will be reported with 95% bootstrap confidence intervals and z-test conducted.

Patient and Public Involvement

WHO's End TB Strategy includes policy goals around elimination of patient catastrophic costs, and this study has been developed to measure and inform both public and stakeholders regarding the economic impact of MDR-TB on patients.

The health economic research questions were developed based on the STOP-TB questionnaire by the health economic team involved in conducting the study at Liverpool School of Tropical Medicine and University of Warwick, based on clinical practice, trial protocol and literature review. All health economic questionnaires have been pilot tested with opportunity for patients to give feedback.

Community advisory boards (CABs), comprised of volunteers from (among others) community-based organizations, those affected by TB, and sometimes trial team members, are functioning with the support of the trial at all 13 STREAM Stage 2 sites. Most CABs were formed at site initiation and, therefore, did not inform the development of the research question and outcome measures; however, input on the trial protocol was received from the Global TB CAB. The STREAM CABs act as coordinating mechanisms for community engagement at STREAM trial sites. Their activities include community outreach (engaging the local communities and key populations to raise awareness and literacy on MDR-TB, research, and the trial), provision of psychosocial support to study patients and advocacy activities aimed at improving programs and policies. The CABs also meet regularly with their respective study teams for trial updates and to pass on patient and community feedback from the trial. Results of the trial will be disseminated to participants and affected communities, with the support of STREAM CABs, likely at outreach events for participants and their families.

The burden of the intervention will be assessed by the patients taking part in the health economic component of the trial, through the EQ-5D-5L questionnaire, which is a self-reported measure of quality of life. These patients will also assess the economic impact the disease had, by reporting changes in income and employment status throughout the study.

COVID-19 impact

Also, the COVID-19 outbreak started during the trial. Lockdown has been imposed on the 18th March in Uganda and on the 24th March in India, while Moldova and Ethiopia declared

For peer review only - http://bmjopen.bmj.com/site/about/guidelines.xhtml

state of emergency in March 2020. It is expected that the COVID19 mitigating measures taken in most countries will affect the socioeconomic status of the patients and their quality of life, independent of their MDR-TB or MDR-TB treatment[14]. There are a few measures that will be taken to record this. A COVID19 diary, containing information about the lockdown restrictions will be completed by each site (see Annex 3). Also, an additional questionnaire has been developed to further explore some of the answers regarding their income, spending and health-related quality of life.

As data collection started in 2016, before the outbreak, the lockdown imposed will be modelled as an independent explanatory variable for parameters such as quality of life, working hours and supplements spending during intensive, continuation and post-treatment phase. If the variable turns out to be significant, we will use it to adjust values reported post-pandemic, using model predictions of what would have been reported if the pandemic hadn't happened.

Additional changes to the protocol as a result of COVID19 may be implemented as needed.

Discussion

STREAM will be the first study to estimate the costs incurred by both patients undergoing MDR-TB treatment and the health care system within a Phase III RCT.

The detailed costing and analysis in four different settings will provide insights into the timing and drivers of the cost saving or dissaving of implementing a nine month all-oral bedaquiline containing regimen, providing the data for targeted interventions if needed.

The study will have certain limitations. The EQ-5D-5L is not a condition-specific measure, and so may miss differences in symptoms that are important to participants. Also, our method assumes a linear relationship between values at different time points, however, this might not be accurate. It was considered not feasible to ask participants to complete the EQ-5D-5L questionnaire at a more frequent interval, i.e. each DOT visit.

The trial setting also means that the experience of participants might be different from routine practice, in ways that could influence costs, such as the frequency of visits and their location and the provision of support (e.g. transport vouchers, food vouchers).

Word count: 3144

Funding

This article is made possible by the generous support of the American people through the United States Agency for International Development (USAID). The contents are the responsibility of the authors and do not necessarily reflect the views of USAID or the United States Government. Stage 2 is funded by the United States Agency for International Development (USAID) and Janssen Pharmaceuticals, with additional funding from the UK Medical Research Council and the UK Department for International Development (DFID), under the USAID Cooperative Agreement GHN-A-00-08-000040-00. It is sponsored by Vital

For peer review only - http://bmjopen.bmj.com/site/about/guidelines.xhtml

Strategies, Inc. (an affiliate of The International Union Against Tuberculosis and Lung Disease).

Competing interests

The authors and collaborating authors declare no competing interests.

Contributorship statement

SBS has obtained research funding, is the principal investigator of the study and contributed to the original design of this economic study. LR drafted the protocol and contributed to the design of this study. JM, EW and ET provided helpful feedback for all aspects of the work, contributed to the design of the study and revised the draft manuscript.

Data sharing statement

Data will be shared using a controlled access approach; a fully anonymized dataset will be made available to qualified researchers through application for access from a public data sharing platform.

STREAM Study Health Economic Evaluation Collaborators

Mamo Girma, Vanita Patel, Makwana Mukesh, Malaisamy Muniyandi, Shravan Kumar, Sangeetha Subramani, Saleem Ahmad, Jasper Nidoi, Irina Pirlog, Mariana Macarie, I.D. Rusen, Gay Bronson, Meera Gurumurthy, Karen Sanders, Sarah Meredith, Andrew Nunn, Ben Spittle, Wendy Dodds, Robyn Henry-Cockles, Rachel Bennett, Elisa Giallongo, Danni Maas, Rachel Bennett, Ruth Goodall, Saiam Ahmed, Claire Cook, Katharine Bellenger, Gopalan Narendran, Bruce Kirenga, Elena Tudor, Rajesh Solanki, Daniel Meressa, Adamu Bayissa, Anuj Bhatnagar, STREAM Community advisory boards (CABs).

REFERENCES

1. World Health Organization. (2018). Global tuberculosis report 2018.
2. World Health Organization. (2014). The End TB Strategy: Global strategy targets for tuberculosis prevention, care and control after 2015.
3. World Health Organization. (2019). Rapid communication: key changes to treatment of drug-resistant tuberculosis.
4. World Health Organization. (2018). Rapid communications: key changes to treatment of multidrug- and rifampicin-resistant tuberculosis (MDR/RR-TB).
5. The STREAM protocol v8.0

For peer review only - http://bmjopen.bmj.com/site/about/guidelines.xhtml

6. Madan, Jason J, Rosu, Laura, Tefera, Mamo Girma, van Rensburg, Craig, Evans, Denise. et al. (2020). Economic evaluation of short treatment for multidrug-resistant tuberculosis, Ethiopia and South Africa: the STREAM trial. Bulletin of the World Health Organization, 98 (5), 306 - 314, http://dx.doi.org/10.2471/BLT.19.243584

7. The Tool to Estimate Patients' Cost. (2008). TB|CTA (http://www.stoptb.org/wg/dots_expansion/tbandpoverty/assets/documents/Tool%20to%20estimate%20Patients'%20Costs.pdf)

8. OANDA.com. Currency Converter (https://www1.oanda.com/currency/converter/)

9. EuroQol. (https://euroqol.org/eq-5d-instruments/eq-5d-5l-about/

10. Woods, B., Revill, P., Sculpher, M., Claxton, K. (2016). Country-Level Cost-Effectiveness Thresholds: Initial Estimates and the Need for Further Research. Value in Health, 19 (8), 929-935, https://doi.org/10.1016/j.jval.2016.02.017

11. Royston P. Multiple imputations of missing values. (2004). The Stata Journal 2004, 3:227-241.

12. ISPOR. Good Practices for Outcomes Research (https://www.ispor.org/heor-resources/good-practices-for-outcomes-research)

13. Andrew A. Anderson (2019) Assessing Statistical Results: Magnitude, Precision, and Model Uncertainty, The American Statistician, 73:sup1, 118-121, DOI: 10.1080/00031305.2018.1537889X.

14. Chirisa, I., Mutambisi, T., Chivenge, M. *et al*. The urban penalty of COVID-19 lockdowns across the globe: manifestations and lessons for Anglophone sub-Saharan Africa. GeoJournal (2020). https://doi.org/10.1007/s10708-020-10281-6

For peer review only

For peer review only - http://bmjopen.bmj.com/site/about/guidelines.xhtml

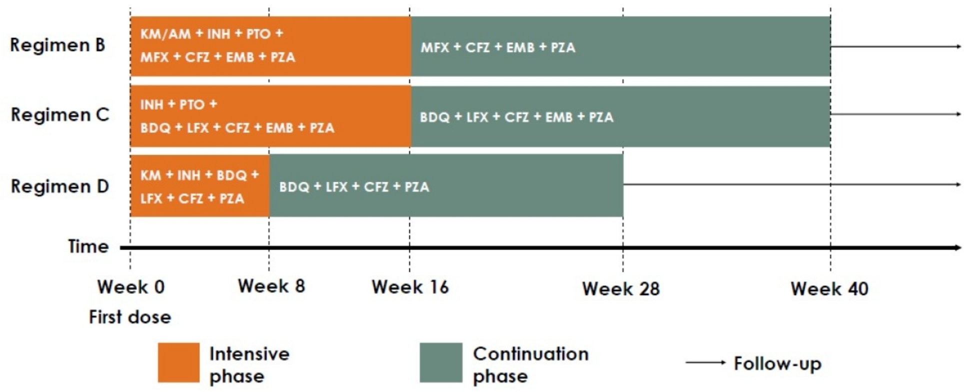

Figure 1 Treatments outline. Regimen A was dropped of the trial.

331x128mm (96 x 96 DPI)

-Supplementary material-

Annex 1- CHEERS Checklist

Section	Item No	Recommendation	Reported on page No/line No
**Title and Abstract**			
**Title**	1	Identify the study as an economic evaluation or use more specific terms such as "cost-effectiveness analysis", and describe the interventions compared.	Title is: Economic evaluation of a short, all-oral bedaquiline-containing regimen for the treatment of rifampicin-resistant tuberculosis from the STREAM trial
**Abstract**	2	Provide a structured summary of objectives, perspective, setting, methods (including study design and inputs), results (including base case and uncertainty analyses), and conclusions.	N/A as it is an analysis plan
**Introduction**			
**Background and objectives**	3	Provide an explicit statement of the broader context for the study.<br><br>Present the study question and its relevance for health policy or practice decisions.	Covered in Background and Objectives sections
**Methods**			
**Target population and subgroups**	4	Describe characteristics of the base case population and subgroups analyzed, including why they were chosen.	Covered in the Methods and Analysis section
**Setting and location**	5	State relevant aspects of the system(s) in which the decision(s) need(s) to be made.	Covered in the Methods and Analysis section
**Study perspective**	6	Describe the perspective of the study and relate this to the costs being evaluated.	Covered in the Methods and Analysis section
**Comparators**	7	Describe the interventions or strategies being compared and state why they were chosen.	Covered in the Methods and Analysis section
**Time horizon**	8	State the time horizon(s) over which costs and consequences are being evaluated and say why appropriate.	Covered in the Methods and Analysis section
**Discount rate**	9	Report the choice of discount rate(s) used for costs and outcomes and say why appropriate.	In the Methods section, Health system resource use and cost sub-heading
**Choice of health outcomes**	10	Describe what outcomes were used as the measure(s) of benefit in the evaluation and their relevance for the type of analysis performed.	Covered in the Methods and Analysis section
**Measurement of effectiveness**	11a	*Single study-based estimates*: Describe fully the design features of the single effectiveness study and why the single study was a sufficient source of clinical effectiveness data.	Reference to the clinical paper; Covered in the Methods and Analysis section
	11b	*Synthesis-based estimates*: Describe fully the methods used for identification of included studies and synthesis of clinical effectiveness data.	N/A

For peer review only

**Measurement and valuation of preference-based outcomes**	12	If applicable, describe the population and methods used to elicit preferences for outcomes.	N/A
**Estimating resources and costs**	13a	*Single study-based economic evaluation*: Describe approaches used to estimate resource use associated with the alternative interventions. Describe primary or secondary research methods for valuing each resource item in terms of its unit cost. Describe any adjustments made to approximate to opportunity costs.	Fully described in Methods and Analysis section
	13b	*Model-based economic evaluation*: Describe approaches and data sources used to estimate resource use associated with model health states. Describe primary or secondary research methods for valuing each resource item in terms of its unit cost. Describe any adjustments made to approximate to opportunity costs.	N/A
**Currency, price date, and conversion**	14	Report the dates of the estimated resource quantities and unit costs. Describe methods for adjusting estimated unit costs to the year of reported costs if necessary. Describe methods for converting costs into a common currency base and the exchange rate.	Dates of the estimated resource quantities and unit costs not reported as this is a protocol. Methods for adjusting the unit costs and converting costs into a common currency are covered in the Methods and Analysis section, after the Patient costs sub-heading.
**Choice of model**	15	Describe and give reasons for the specific type of decision analytical model used. Providing a figure to show model structure is strongly recommended.	N/A as not a model-based evaluation
**Assumptions**	16	Describe all structural or other assumptions underpinning the decision-analytical model.	N/A as not a model-based evaluation
**Analytical methods**	17	Describe all analytical methods supporting the evaluation. This could include methods for dealing with skewed, missing, or censored data; extrapolation methods; methods for pooling data; approaches to validate or make adjustments (such as half cycle corrections) to a model; and methods for handling population heterogeneity and uncertainty.	Fully covered in the Methods and Analysis section, in the Missing data, Statistical analysis and Sensitivity analyses sub-sections.
**Results**			
**Study parameters**	18	Report the values, ranges, references, and, if used, probability distributions for all parameters. Report reasons or sources for distributions used to represent uncertainty where appropriate. Providing a table to show the input values is strongly recommended.	N/A as this is a study protocol, but these will be presented in the main paper as stated in this protocol
**Incremental costs and outcomes**	19	For each intervention, report mean values for the main categories of estimated costs and outcomes of interest, as well as mean differences between the comparator groups. If applicable, report incremental cost-effectiveness ratios.	N/A as this is a study protocol, but these will be presented in the main paper as stated in this protocol

**Characterizing uncertainty**	**20a**	*Single study-based economic evaluation*: Describe the effects of sampling uncertainty for the estimated incremental cost and incremental effectiveness parameters, together with the impact of methodological assumptions (such as discount rate, study perspective).	Methods and Analysis section of the protocol-Sensitivity analyses sub-heading.
	20b	*Model-based economic evaluation*: Describe the effects on the results of uncertainty for all input parameters, and uncertainty related to the structure of the model and assumptions.	N/A
**Characterizing heterogeneity**	21	If applicable, report differences in costs, outcomes, or cost-effectiveness that can be explained by variations between subgroups of patients with different baseline characteristics or other observed variability in effects that are not reducible by more information.	Costs and outcomes will be presented separately for each country

For peer review only

Discussion			
**Study findings, limitations, generalizability, and current knowledge**	22	Summarise key study findings and describe how they support the conclusions reached. Discuss limitations and the generalizability of the findings and how the findings fit with current knowledge.	Discussion about the strengths and limitations in the Discussion section; the key findings and their generalizability will be presented in the paper.
**Other**			
**Source of funding**	23	Describe how the study was funded and the role of the funder in the identification, design, conduct, and reporting of the analysis. Describe other non-monetary sources of support.	Acknowledgements
**Conflicts of interest**	24	Describe any potential for conflict of interest of study contributors in accordance with journal policy. In the absence of a journal policy, we recommend authors comply with International Committee of Medical Journal Editors recommendations.	Acknowledgements

Annex 2- Value sets to be used

For peer review only

Independent variables of the model	C-TTO Tobit model censored at −1			DCE conditional logistic model rescaled			Hybrid model censored C-TTO values at −1 (final value set)		
	Coeff.	(SE)	*p* value	Coeff.	(SE)	*p* value	Coeff.	(SE)	*p* value
Mobility (MO)									
No problems to slight problems	0.088	(0.015)	0.000	0.139	(0.015)	0.000	0.119	(0.008)	0.000
Slight problems to moderate problems	0.086	(0.017)	0.000	0.080	(0.017)	0.000	0.073	(0.011)	0.000
Moderate problems to severe problems	0.250	(0.019)	0.000	0.196	(0.016)	0.000	0.218	(0.013)	0.000
Severe problems to unable	0.170	(0.018)	0.000	0.219	(0.018)	0.000	0.203	(0.012)	0.000
Self-care (SC)									
No problems to slight problems	0.085	(0.014)	0.000	0.101	(0.016)	0.000	0.101	(0.007)	0.000
Slight problems to moderate problems	0.056	(0.018)	0.002	0.038	(0.018)	0.032	0.039	(0.010)	0.000
Moderate problems to severe problems	0.128	(0.018)	0.000	0.085	(0.019)	0.000	0.108	(0.013)	0.000
Severe problems to unable	0.035	(0.016)	0.030	0.097	(0.017)	0.000	0.068	(0.012)	0.000
Usual activities (UA)									
No problems to slight problems	0.071	(0.015)	0.000	0.092	(0.016)	0.000	0.090	(0.006)	0.000
Slight problems to moderate problems	0.106	(0.017)	0.000	0.051	(0.017)	0.003	0.066	(0.011)	0.000
Moderate problems to severe problems	0.137	(0.019)	0.000	0.154	(0.017)	0.000	0.145	(0.013)	0.000
Severe problems to unable	0.061	(0.018)	0.001	0.091	(0.017)	0.000	0.084	(0.013)	0.000
Pain/discomfort (PD)									
No problems to slight problems	0.089	(0.013)	0.000	0.081	(0.016)	0.000	0.086	(0.006)	0.000
Slight problems to moderate problems	0.007	(0.019)	0.721	0.012	(0.018)	0.513	0.009	(0.011)	0.395
Moderate problems to severe problems	0.135	(0.018)	0.000	0.085	(0.017)	0.000	0.103	(0.013)	0.000
Severe problems to extreme problems	0.024	(0.019)	0.211	0.053	(0.018)	0.003	0.048	(0.013)	0.000
Anxiety/depression (AD)									
No problems to slight problems	0.079	(0.014)	0.000	0.050	(0.017)	0.003	0.079	(0.006)	0.000
Slight problems to moderate problems	0.055	(0.018)	0.002	0.061	(0.017)	0.000	0.055	(0.011)	0.000
Moderate problems to severe problems	0.086	(0.017)	0.000	0.114	(0.018)	0.000	0.093	(0.012)	0.000
Severe problems to extreme problems	0.062	(0.016)	0.000	0.085	(0.018)	0.000	0.078	(0.012)	0.000
Log likelihood	−6189.97			−3958.62			−9325.84		
AIC	12,421.93			7957.24			18,735.69		
BIC	12,572.19			8109.23			19,060.41		
Examples of estimated utility values									
U(21111)	0.912			0.861			0.881		
U(31111)	0.826			0.781			0.808		
U(41111)	0.576			0.585			0.590		
U(51111)	0.406			0.366			0.387		
U(12345)	0.225			0.268			0.240		
U(21231)	0.745			0.676			0.696		
U(55555)	−0.810			−0.884			−0.865		

AIC Akaike information criteria, *BIC* Bayesian information criteria, *C-TTO* composite time trade-off, *DCE* discrete choice experiments, SE standard error

Table 1- Value set to be used for India. Purba FD, Hunfeld JAM, Iskandarsyah A, et al. The Indonesian EQ-5D-5L Value Set. Pharmacoeconomics. 2017;35(11):1153-1165. doi:10.1007/s40273-017-0538-9

Independent variables of the model	C-TTO OLS model			DCE conditional logistic model rescaled			Hybrid model censored C-TTO values at -1 (final value set)		
	Coef.	(SE)	p-value	Coef.	(SE)	p-value	Coef.	(SE)	p-value
**Mobility (MO)**									
MO2	0.0047	0.014	0.729	0.4780	0.061	0.000	0.0337	0.005	0.000
MO3	0.0166	0.015	0.262	0.1138	0.071	0.110	0.0307	0.009	0.000
MO4	0.1748	0.016	0.000	0.9810	0.070	0.000	0.1632	0.010	0.000
MO5	0.1038	0.016	0.000	0.7434	0.074	0.000	0.1322	0.010	0.000
**Self-care (SC)**									
SC2	0.0036	0.013	0.785	0.2044	0.067	0.002	0.0235	0.005	0.000
SC3	0.0494	0.016	0.002	-0.0024	0.074	0.974	0.0160	0.008	0.042
SC4	0.1189	0.015	0.000	0.6849	0.078	0.000	0.1024	0.009	0.000
SC5	0.0826	0.013	0.000	0.4234	0.073	0.000	0.0804	0.009	0.000
**Usual-activities (UA)**									
UA2	0.0188	0.014	0.176	0.3470	0.063	0.000	0.0323	0.005	0.000
UA3	0.0441	0.014	0.002	-0.0391	0.071	0.579	0.0160	0.008	0.042
UA4	0.1299	0.016	0.000	0.5818	0.071	0.000	0.1091	0.009	0.000
UA5	0.0936	0.015	0.000	0.6079	0.076	0.000	0.1147	0.010	0.000
**Pain/discomfort (PD)**									
PD2	0.0140	0.013	0.266	0.4499	0.067	0.000	0.0361	0.004	0.000
PD3	0.0161	0.017	0.331	0.1090	0.073	0.136	0.0155	0.008	0.061
PD4	0.2452	0.015	0.000	1.1358	0.077	0.000	0.2187	0.010	0.000
PD5	0.1421	0.016	0.000	0.5689	0.076	0.000	0.1361	0.011	0.000
**Anxiety/depression (AD)**									
AD2	0.0111	0.014	0.428	0.2718	0.070	0.000	0.0259	0.004	0.000
AD3	0.0381	0.015	0.012	0.3516	0.072	0.000	0.0589	0.008	0.000
AD4	0.2322	0.015	0.000	1.1803	0.079	0.000	0.2139	0.009	0.000
AD5	0.1414	0.013	0.000	0.8320	0.078	0.000	0.1591	0.010	0.000
**AIC**	10587.06			6498.30			14002.09		
**BIC**	10739.33			6650.17			14336.81		
**Order of importance**									
	AD			AD			AD		
	PD			MO			PD		
	MO			PD			MO		
	UA			UA			UA		
	SC			SC			SC		

Coef. – coefficient; SE – standard error

Items with a negative coefficient (in grey) represent inconsistent items
Order of importance based on sum of disutility which is the disutility associated with level 5

Table 2- Value set to be used for Uganda and Ethiopia. Welie AG, Gebretekle GB, Stolk E, Mukuria C, Krahn MD, Enquoselassie F, Fenta TG. Valuing health state: an EQ-5D-5L value set for Ethiopians. Value Health Reg Issues. 2019;22:7–14

	Model 1 panel, random effects	Model 2 Bayesian	Model 3 M2 + random parameters	Model 4 M3 + error scaling with *t*-Student	Model 5 M4 + religion scaling	Final model M5 + DCE, censoring
Const.	0.005 (−0.010; 0.019)	Not used	Not used	Not used	Not used	Not used
MO2	0.021 (0.002; 0.039)	0.023 (0.001; 0.044)	0.058 (0.013; 0.073)	0.017 (0.014; 0.022)	0.019 (0.014; 0.023)	0.025 (0.020; 0.029)
MO3	0.012 (−0.007; 0.031)	0.016 (0.000; 0.036)	0.077 (0.021; 0.094)	0.015 (0.005; 0.026)	0.016 (0.005; 0.028)	0.034 (0.026; 0.042)
MO4	0.098 (0.077; 0.118)	0.101 (0.074; 0.129)	0.159 (0.071; 0.181)	0.101 (0.085; 0.116)	0.107 (0.090; 0.124)	0.126 (0.113; 0.141)
**MO5**	**0.262 (0.238; 0.285)**	**0.263 (0.239; 0.289)**	**0.303 (0.271; 0.330)**	**0.251 (0.228; 0.274)**	**0.267 (0.242; 0.293)**	**0.314 (0.286; 0.342)**
SC2	0.030 (0.014; 0.046)	0.037 (0.015; 0.059)	0.015 (0.003; 0.087)	0.029 (0.024; 0.034)	0.031 (0.026; 0.036)	0.031 (0.027; 0.036)
SC3	0.038 (0.017; 0.059)	0.042 (0.014; 0.071)	0.005 (0.000; 0.119)	0.037 (0.028; 0.047)	0.040 (0.029; 0.050)	0.047 (0.040; 0.055)
SC4	0.122 (0.098; 0.146)	0.116 (0.089; 0.143)	0.042 (0.027; 0.180)	0.108 (0.094; 0.123)	0.115 (0.099; 0.131)	0.111 (0.099; 0.123)
**SC5**	**0.276 (0.254; 0.298)**	**0.269 (0.244; 0.295)**	**0.242 (0.193; 0.268)**	**0.258 (0.237; 0.282)**	**0.273 (0.249; 0.299)**	**0.264 (0.243; 0.286)**
UA2	0.031 (0.014; 0.048)	0.034 (0.011; 0.058)	0.002 (0.000; 0.007)	0.033 (0.026; 0.039)	0.034 (0.028; 0.042)	0.023 (0.019; 0.027)
UA3	0.032 (0.009; 0.054)	0.041 (0.015; 0.067)	0.005 (0.000; 0.014)	0.050 (0.040; 0.060)	0.053 (0.043; 0.063)	0.040 (0.032; 0.048)
UA4	0.092 (0.070; 0.115)	0.088 (0.062; 0.115)	0.024 (0.010; 0.038)	0.104 (0.091; 0.117)	0.110 (0.095; 0.125)	0.097 (0.087; 0.107)
**UA5**	**0.186 (0.167; 0.206)**	**0.183 (0.157; 0.209)**	**0.180 (0.161; 0.201)**	**0.180 (0.161; 0.200)**	**0.190 (0.169; 0.212)**	**0.205 (0.188; 0.224)**
PD2	0.028 (0.012; 0.044)	0.033 (0.012; 0.054)	0.041 (0.028; 0.054)	0.025 (0.021; 0.028)	0.026 (0.022; 0.030)	0.030 (0.026; 0.034)
PD3	0.034 (0.014; 0.053)	0.035 (0.007; 0.063)	0.053 (0.036; 0.071)	0.030 (0.022; 0.039)	0.032 (0.022; 0.041)	0.050 (0.043; 0.058)
PD4	0.229 (0.208; 0.251)	0.228 (0.204; 0.254)	0.253 (0.224; 0.276)	0.223 (0.208; 0.239)	0.235 (0.217; 0.253)	0.261 (0.244; 0.280)
**PD5**	**0.467 (0.440; 0.494)**	**0.473 (0.446; 0.499)**	**0.490 (0.464; 0.518)**	**0.492 (0.463; 0.520)**	**0.519 (0.485; 0.555)**	**0.575 (0.538; 0.613)**
AD2	0.024 (0.006; 0.041)	0.032 (0.010; 0.054)	0.049 (0.015; 0.061)	0.019 (0.016; 0.023)	0.020 (0.017; 0.024)	0.018 (0.015; 0.021)
AD3	0.034 (0.011; 0.056)	0.033 (0.006; 0.058)	0.085 (0.038; 0.101)	0.037 (0.026; 0.049)	0.039 (0.027; 0.052)	0.029 (0.022; 0.037)
AD4	0.114 (0.094; 0.135)	0.114 (0.088; 0.139)	0.160 (0.116; 0.181)	0.119 (0.106; 0.132)	0.126 (0.113; 0.142)	0.108 (0.097; 0.119)
**AD5**	**0.224 (0.203; 0.244)**	**0.226 (0.201; 0.251)**	**0.176 (0.153; 0.231)**	**0.211 (0.194; 0.229)**	**0.223 (0.204; 0.243)**	**0.232 (0.213; 0.252)**
Deviance	61.2% (R^2 used instead)	11,866	−777	−13,781	−13,780	−9215
DIC		11,886	2597	−9704	−9704	−9215[a]
PSRF	n.a.	All <1.01	Maximum = 15	All <1.01	All <1.01	All <1.01
Maximum *u* (not 11111)	0.983	0.984	0.998	0.985	0.984	0.982
u (22222)	0.862	0.841	0.834	0.877	0.870	0.873
u (33333)	0.847	0.833	0.775	0.830	0.821	0.800
u (44444)	0.340	0.352	0.361	0.345	0.307	0.296
u (55555)	− 0.420	− 0.415	− 0.391	− 0.392	− 0.471	− 0.590
% states $u < 0$	2.85	2.88	2.69	2.78	4.26	6.66
Dimension order	PD, SC, MO, AD, UA	PD, SC, MO, AD, UA	PD, MO, SC, UA, AD	PD, SC, MO, AD, UA	PD, SC, MO, AD, UA	PD, MO, SC, AD, UA
Levels consistency	MO3 < MO2	MO3 < MO2	SC3 < SC2	MO3 < MO2	MO3 < MO2	Consistent

AD anxiety/depression, *DCE* discrete choice experiment, *DIC* deviance information criterion, *M* model, *MO* mobility, *n.a.* *PD* pain/discomfort, *PSRF* potential scale reduction factor, *SC* self-care, *u* utility, *UA* usual activities

[a]Failed to calculate penalty in JAGS ("support of observed nodes is not fixed")

Table 3- Value set to be used for Moldova. Golicki, D., Jakubczyk, M., Graczyk, K. et al. Valuation of EQ-5D-5L Health States in Poland: the First EQ-VT-Based Study in Central and Eastern Europe. PharmacoEconomics 37, 1165–1176 (2019). https://doi.org/10.1007/s40273-019-00811-7

Annex 3- COVID19 diary

<u>COVID19 diary</u>

<u>-to be completed by focal health economists at each site-</u>

Epidemiology of the Epidemic

- First case notification date

Details of policies declared by central/federal/state government that potentially restrict "Normal" daily life. Date implemented/Details of policy/Date lifted

- Lockdown start date
- Specific restrictions- what's the rule of going outside the house? What's the rule for going out for work?
- Law enforcement- are people being fined for going out?

Only

- Are entertainment places open (cinemas, theatres shopping centres)? Are cricket, football, etc. competitions still taking place? If not, when were these stopped?
- Lockdown end date

Impact on daily life (descriptive/opinion) behavioural picture

- Country's general perception regarding COVID19- are they scared, complaint with the rules, are they indifferent
- Can you find basic supplies in the markets/supermarkets? Rice, bread? Is there a price increase amongst basic supplies?
- Are people living with their families during the lockdown? Have they travelled to their home town/village during the lockdown?
- Any shortage in drug supplies?
- Anything else you would like to report, that would influence the patients' income and their quality of life?

For peer review only
